# Efficient Chemical-Free Degradation of Waterborne Micropollutants with an Immobilized Dual-Porous TiO_2_ Photocatalyst

**DOI:** 10.1021/acsestengg.3c00191

**Published:** 2023-08-03

**Authors:** Daniel E. Willis, Ella C. Sheets, Mary R. Worbington, Madhusudan Kamat, Sarah K. Glass, MaCayla J. Caso, Tochukwu Ofoegbuna, Liz M. Diaz, Caleb Osei-Appau, Samuel D. Snow, Kevin M. McPeak

**Affiliations:** † Gordon and Mary Cain Department of Chemical Engineering, Louisiana State University, Baton Rouge, Louisiana 70803, United States; § Department of Civil and Environmental Engineering, Louisiana State University, Baton Rouge, Louisiana 70803, United States

**Keywords:** Advanced Oxidation, Water Treatment, Water Reuse, Photocatalysis, Micropollutants

## Abstract

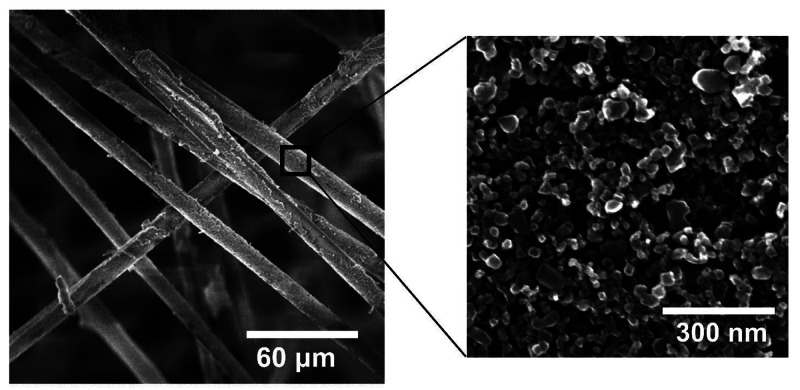

Photocatalytic advanced oxidation processes (AOPs) promise a chemical-free route to energy-efficient degradation of waterborne micropollutants if long-standing mass transfer and light management issues can be overcome. Herein, we developed a dual-porous photocatalytic system consisting of a mesoporous (i.e., 2–50 nm pores) TiO_2_ (P25) photocatalyst supported on macroporous (i.e., >50 nm pores) fused quartz fibers (P25/QF). Our reusable photocatalytic AOP reduces chemical consumption and exhibits excellent energy efficiency, demonstrated by degrading various pharmaceutical compounds (acetaminophen, sulfamethoxazole, and carbamazepine) in natural waters with electrical energy per order (E_EO_) values of 4.07, 0.96, and 1.35 kWh/m^3^, respectively. Compared to the conventional H_2_O_2_/UVC AOP, our photocatalytic AOP can treat water without chemical additives while reducing energy consumption by over 2800%. We examine these improvements based on mass transport and optical (UVA and UVC) transmittance and demonstrate that the enhancements scale with increasing flow rate.

## 1 Introduction

Water scarcity is a growing challenge for nations, rich and poor. Reclaiming and reusing freshwater helps maintain precious resources in drought-prone areas such as the Western United States and Israel.^1^ Unfortunately, potable water reuse for even small municipalities can cost millions of USD per year.^2,3^ Approximately 10% of potable reuse costs are tied to tertiary water treatment, such as advanced oxidation processes (AOPs), with nearly 40% of operations and maintenance costs for UV-driven AOP systems attributed to the consumption of chemical additives (e.g., H_2_O_2_).^3,4^ Reducing energy and chemical requirements is paramount for sustainable water treatment/reuse strategies.

Photocatalytic AOPs offer potential advantages over conventional AOPs through (1) the ability to use lower energy photons, (2) the presence of catalytic surfaces for contaminant adsorption and direct breakdown, and (3) the potential cost savings by eliminating the sourcing, dosing, and quenching of chemical additives.^5,6^ However, previous photocatalytic technologies have largely fallen short of commercial application for several reasons. The few existing cases of commercialized photocatalytic treatment implement TiO_2_ particles in suspension, taking advantage of the high catalyst surface area with minimal mass transport limitations. However, these slurry-type reactors are limited by the depth of UV penetration and require the filtration of suspended catalyst particles from the treated effluent.^5,7−9^ Immobilizing the photocatalyst on a flat surface makes catalyst filtration unnecessary but severely limits mass transport.^10,11^ Several works have attempted to resolve both issues by immobilizing the photocatalyst on fibrous supports made of glass or plastic.^11−13^ While these supporting materials are transparent to visible and UVA light, they act as parasitic absorbers in the UVC region. Furthermore, while visible and UVA driven photocatalytic studies are abundant in the literature, current municipal and industrial water treatment systems employ UVC for disinfection and AOPs.^7,9,14^ Adaptation of extant treatment systems to use photocatalysis should be designed for UVC irradiation. Studies that use UVC with TiO_2_ are limited but promising.^15−20^

In this work, we explore the use of a novel photocatalyst support system to improve process efficiency by fabricating and evaluating a porous TiO_2_ film attached to UV-transparent quartz fibers (QF). We find, theoretically and experimentally, that this dual-porous system of mesoporous P25 films on macroporous fiber support offers benefits over comparative supported photocatalysts within this work and from previous studies due to increased surface area, improved mass transport, and better light management.^21−25^ We demonstrate the impact of porosity through the comparative generation of hydroxyl radicals, ·OH, and resultant degradation of common probe molecules from various chemical classes, including organic acids (terephthalic acid, TPA), dyes (Rhodamine B, RhB), alcohols (furfuryl alcohol, FFA), and aromatics (4-chlorophenol, 4-CP) under commercially relevant, near-neutral pH conditions. As a figure-of-merit for the commercial potential of our dual-porous photocatalytic AOP, we calculate the electrical energy per order (E_EO_) for the degradation of the probe molecules and compare the E_EO_ values to those obtained using a traditional H_2_O_2_/UVC AOP.^26,27^ To better understand the performance of our dual-porous photocatalyst system, we investigate the impact of the QF support density on the efficiency of the system and explore the role of UV excitation, in terms of both intensity and wavelength, by using a UVA LED—a proposed next-generation light source—and a low-pressure UVC bulb—a current commercial standard.^28^ Finally, we use our dual-porous photocatalyst to degrade pharmaceutical compounds in a river water source with nearly 30 times the treatment efficacy of traditional H_2_O_2_/UVC AOPs. Our P25/QF photocatalyst shows promise for lowering the cost of AOPs for various water treatment applications.

## 2 Experimental Section

### 2.1 Chemicals Used

The following chemicals and materials were used for this work: AEROXIDE TiO_2_ P25 (Evonik); acetylacetone (Alfa Aesar); polyethylene glycol, MW 20,000 (Alfa Aesar); Triton X-100 (Acros Organics); hydrochloric acid (VWR); sulfuric acid (Fisher Scientific); pure ethanol (Koptec); terephthalic acid (Tokyo Chemical Industry); 2-hydroxyterephthalic acid (Ark Pharma); sodium bicarbonate (VWR); calcium chloride (VWR); humic acids (Alfa Aesar); Rhodamine B (Tokyo Chemical Industry); red food dye (McCormick Culinary); acetaminophen (Sigma-Aldrich); sulfamethoxazole (Sigma-Aldrich); carbamazepine (Alfa Aesar); 4-chlorophenol (Sigma-Aldrich); and furfuryl alcohol (VWR). HPLC grade solvents of acetonitrile and methanol were obtained from VWR.

### 2.2 Photocatalyst Fabrication

QF sheets were obtained from Saint-Gobain Quartz (U.S.A.) as Quartzveil sheets and were cut to size (typically 5 cm × 5 cm). The Quartzveil sheets came pretreated with an epoxy binder to help retain their shape. The epoxy binder absorbs in the UV and therefore needed to be removed to avoid parasitic absorption. To remove the epoxy binder, we heated the QF veils in a tube furnace at 500 °C open to the atmosphere for 2 h to combust the organics. Thermogravimetric analysis (TGA) and UV transmittance verified that the epoxy was removed, as shown in Figures S1 and S2 of the Supporting Information. Notably, we did not observe a significant loss in the structural integrity of the QF veil post binder removal.

A TiO_2_ paste was synthesized using Evonik P25 TiO_2_ powder in an aqueous suspension with acetylacetone, PEG, and Triton X-100 as organic binder, spacer, and surfactant, respectively.^29,30^ A P25 paste was synthesized by adding the following to 48 mL of deionized (DI) water, sequentially and slowly, under vigorous mixing: 0.96 mL acetylacetone (i.e., 2,4-pentanedione, an organic stabilizer), 4.8 g PEG (20,000 MW, an emulsifier and void spacer) until dissolved, 12.0 g P25 powder, and 1 drop (∼20 μL) of Triton X-100 (surfactant). After vigorous mixing to homogeneity, the P25 paste was sonicated at 35 kHz for 15 min to further disperse the P25 within the paste. The P25 paste could be stored and reused for up to 1 month, provided it was sonicated or mixed for at least 30 min prior to use. To fabricate P25 films on glass, Eagle XG substrates were dipped into the P25 paste and held for 30 s before removal with a dip-coater at a withdrawal rate of 20 mm/min. Samples were then dried in air for 10 min and annealed in a tube furnace at 550 °C for 2 h with a 5 °C/min ramp rate and cooled to room temperature overnight. When desired, thicker P25 films were fabricated by heating the samples in air at 200 °C after air drying between dip-coating steps. When using the P25 paste to coat QF, the paste was diluted 10× with DI water immediately before use for P25/QF catalysts and was diluted 100× with DI water for dil-P25/QF catalysts. The QF veils were then dip-coated with the same procedure as glass substrates. After coating, the samples were dried by hanging them in air for 1 h before annealing coated samples in the tube furnace at 550 °C for 2 h.

### 2.3 Photocatalyst Characterization

The QF veils were characterized by measuring the average fiber diameter by using optical microscopy, calculating the substrate surface area, and validating the sample density through mass changes. These measurements and calculations are provided in the Supporting Information. QF with a 50 g/m^2^ density was used for most studies, except in section 3.3 where the effect of catalyst loading was investigated by using QF with additional densities of 25 and 10 g/m^2^. The P25/QF system was further characterized via X-ray diffraction (XRD), UV–vis spectroscopy, scanning electron microscopy (SEM), and Brunauer–Emmett–Teller (BET) surface analysis. Details of these processes are provided in the Supporting Information.

Side-view SEM was used to measure the porous P25 film thickness (∼1.8 μm), and eq 1 was used to calculate a mesoporous void fraction of 0.59 within the P25 film (Figure S3):
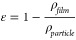
1where ε is the void fraction, ρ_film_ is the measured density of our P25 film, and ρ_particle_ is the density of Evonik P25 nanoparticles (as provided by Evonik). In the experimental studies, P25/glass samples were used with 3 layers of the P25 film applied sequentially and dried at 200 °C in between each layer addition. The 3-layer P25/glass samples absorb >62% of UVA (365 nm) light across ∼5 μm thick films. Note that the P25 titania was unchanged by annealing samples at 550 °C from its original 80% anatase, 20% rutile composition, thus preserving the catalytically active heterojunction.^31−33^

### 2.4 Characterization of Photocatalytic Performance

Photocatalytic experiments were performed at pH 6.5, unless otherwise specified, and in a continuous flow reaction system using a custom-built milliflow reactor (MFR), an in-line spectrofluorometer, and two different UV sources. The MFR (Figure S4) has a 25 cm^2^ quartz window for photoexcitation and provides plug-like fluid flow of up to 5 mL/min through a rectangular channel of 5 cm × 5 cm × 0.05 cm height (1.25 cm^3^). Residence time distributions confirming plug-like flow for the MFR were simulated and experimentally validated (Figure S5). Two UV illumination sources were used in this study: a UVA LED (Waveform, realUV LED Flood Light, 365 nm), with a peak spectral output 365 nm wavelength and an intensity tuned to 3.5 mW/cm^2^, and a UVC low-pressure mercury bulb array (UVP XX-15S) outputting >80% spectral intensity at 254 nm, measured at 5 mW/cm^2^. Intensity values were chosen to provide an equivalent photonic flux of ∼1.6 × 10^17^ photons per second for our system. Millipore 18.2 MΩ-cm DI water was used for all photochemical experiments except when synthetic source water (SSW) or filtered Mississippi River water (MRW) is stated. MRW was collected (30.412572, −91.198142), filtered through a 0.45 μm polypropylene membrane filter prior to analysis, and stored in the dark at 4 °C until use.

Quantification of ·OH generation was performed by observing the selective oxidation of TPA into the fluorescent product hTPA.^34,35^ A concentrated stock TPA solution was prepared by adding powdered TPA into water, adjusting the pH up to neutral (7) with 0.1 M NaOH, and stirring the solution at 50 °C for 12 h to fully dissolve the TPA. This stock TPA solution was diluted to 500 μM and adjusted to pH 7, then used as the test solution for ·OH generation. After passing through the reactor, the solution entered a flow through cuvette mounted on a fluorometer (PTI QM 40). We excited hTPA with 350 nm light and measured its emission intensity at 425 nm to quantify the hTPA concentration. Previous studies approximated the reaction yield of hTPA in the reaction between ·OH and TPA at ∼30%, which we used to estimate the concentration of ·OH formed here.^35^ Additional details of the control experiments for TPA oxidation under UV excitation are provided in the Supporting Information.

The degradation of RhB was monitored via changes in the absorbance at 554 nm. To test the destruction of pharmaceutical species, 4-CP, and FFA, we used an Agilent 1260 Infinity high performance liquid chromatography (HPLC) system with a UV detector. The pharmaceutical samples were run for 12 min using a solvent gradient of acetonitrile/water (phosphoric acid solution) from 30:70 at min 0 to 100:0 at min 7 before returning to 30:70 at min 10. The compounds were analyzed at 280, 254, and 220 nm for acetaminophen, sulfamethoxazole, and carbamazepine, respectively. The degradation of FFA and 4-CP were monitored by HPLC using similar methods without a solvent gradient; FFA was detected at 218 nm with an acetonitrile/water ratio of 30:70, while 280 nm and an acetonitrile/water ratio of 55:45 was used for 4-CP. Initial concentrations for each contaminant were 10 μM of RhB, 10 μM for each pharmaceutical compound, 100 μM of 4-CP, and 500 of μM FFA. To compare our photocatalytic system to a conventional UVC/H_2_O_2_ AOP, we used the same initial concentrations of each contaminant in addition to 6 ppm of H_2_O_2_ at a flow rate of 4.6 mL/min.

The E_EO_ metric is an essential tool for assessing the viability of AOPs for water treatment.^26,27^ E_EO_ quantifies the energy efficiency of a treatment technology scaled over large volumes for a given contaminant removal. In general, an E_EO_ < 10 kWh/m^3^/order is competitive for drinking water applications.^5,27^ For our continuous flow system, the E_EO_ was calculated by
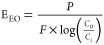
2where *P* is the radiant power from the UV source (kW), *F* is the volumetric flow rate (m^3^/h), and *C*_0_ and *C*_*i*_ are the concentrations of pollutant at the inlet and outlet of the reactor, respectively.

## 3 Results and Discussion

### 3.1 Fabrication and Characterization of Immobilized Photocatalysts

We fabricated and tested three immobilized photocatalyst systems, (1) a microporous film on a flat substrate (P25/glass), (2) a dual-porous system having a microporous TiO_2_ coating on macroporous QF (P25/QF), and (3) a dual-porous system with a reduced catalyst load (dil-P25/QF), and compared them against a traditional UVC/H_2_O_2_ AOP. All our immobilized systems used the same P25 photocatalyst, which was applied to the supports under similar conditions and photoactive under UV radiation >3.2 eV (<385 nm). This allows us to assume similar photocatalytic activity across the three systems and understand their performance differences based on changes in surface area, UV absorption and mass transfer. The P25/glass samples, and several similar designs reported elsewhere,^7,10,22,36−40^ are mass transfer limited due to a low active surface-to-volume ratio, interporous diffusion constraints and poor photon management. These pitfalls motivate the pursuit of multifunctional materials to solve both mass transfer and photon management challenges simultaneously.

**Figure 1:**
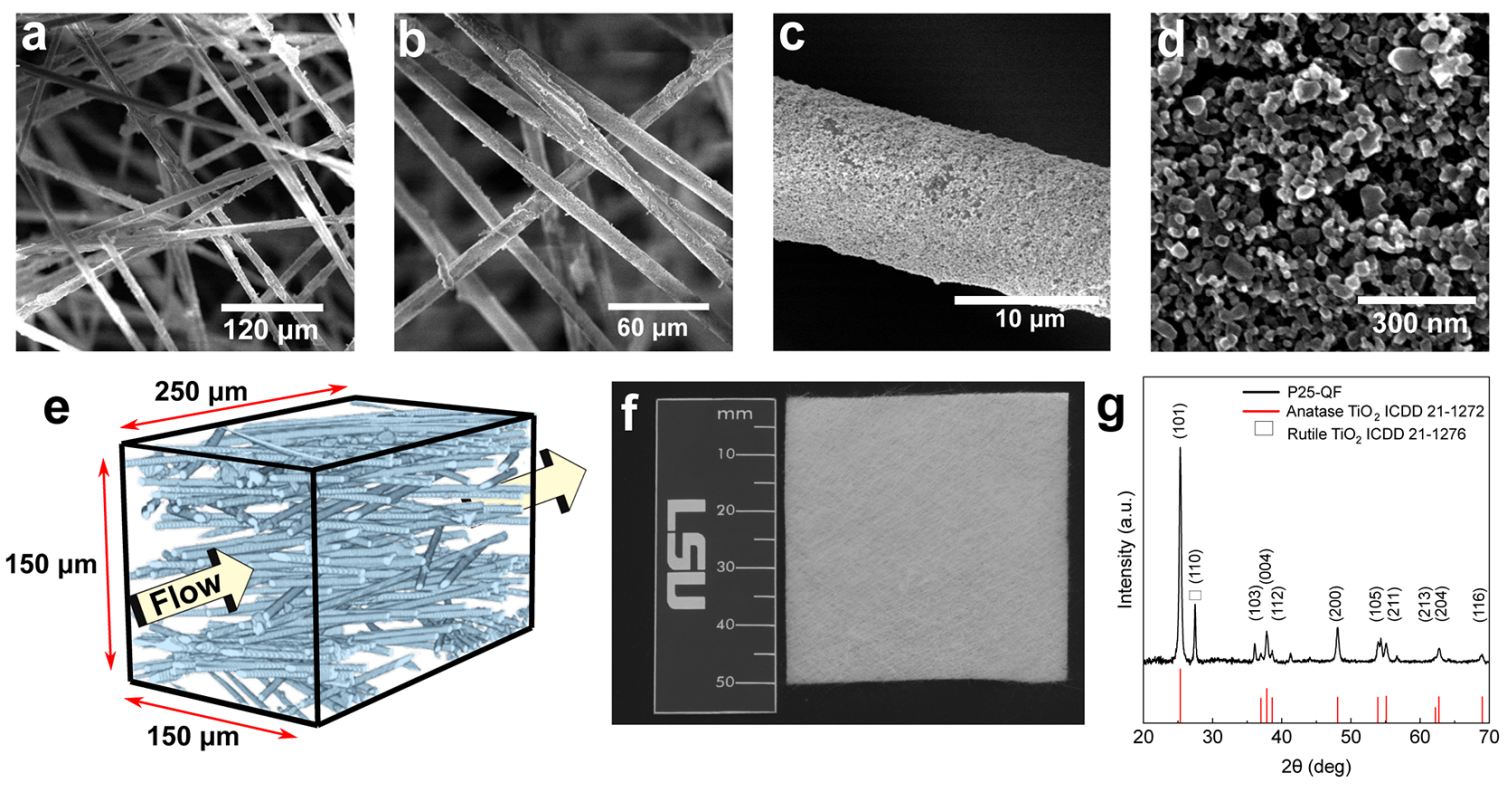
(a–c) Electron micrographs of the P25/QF photocatalyst at varying magnifications and (d) an electron micrograph of the P25/glass surface, along with (e) an X-ray tomograph of the P25/QF structure, with arrows depicting the direction of fluid flow relative to the fibers. (f) Photograph of the 50 mm × 50 mm P25/QF photocatalyst at macroscale. (g) X-ray diffraction scan for the P25/QF sample showing a mixed anatase/rutile titania phase.

We sought to improve both mass transport in aqueous flow and UV photon management by applying a P25 paste to a sample of bare QF (following the same dip-coating and sintering procedures used for P25/glass samples) to create dual-porous (P25/QF) samples. Electron micrographs revealed the micron and nanoscale structures of P25/QF and P25/glass (Figures 1a–c and d, respectively), while X-ray tomographs (Figure 1e) show the random orientation of the ∼6 μm diameter QF. Rectangular sheets of P25/QF were cut with dimensions of 5 cm × 5 cm × 0.05 cm (Figure 1f) for use in the custom-made MFR. XRD analysis (Figure 1g) confirmed that the crystal structure of P25 was a mixture of anatase and rutile.

**Figure 2:**
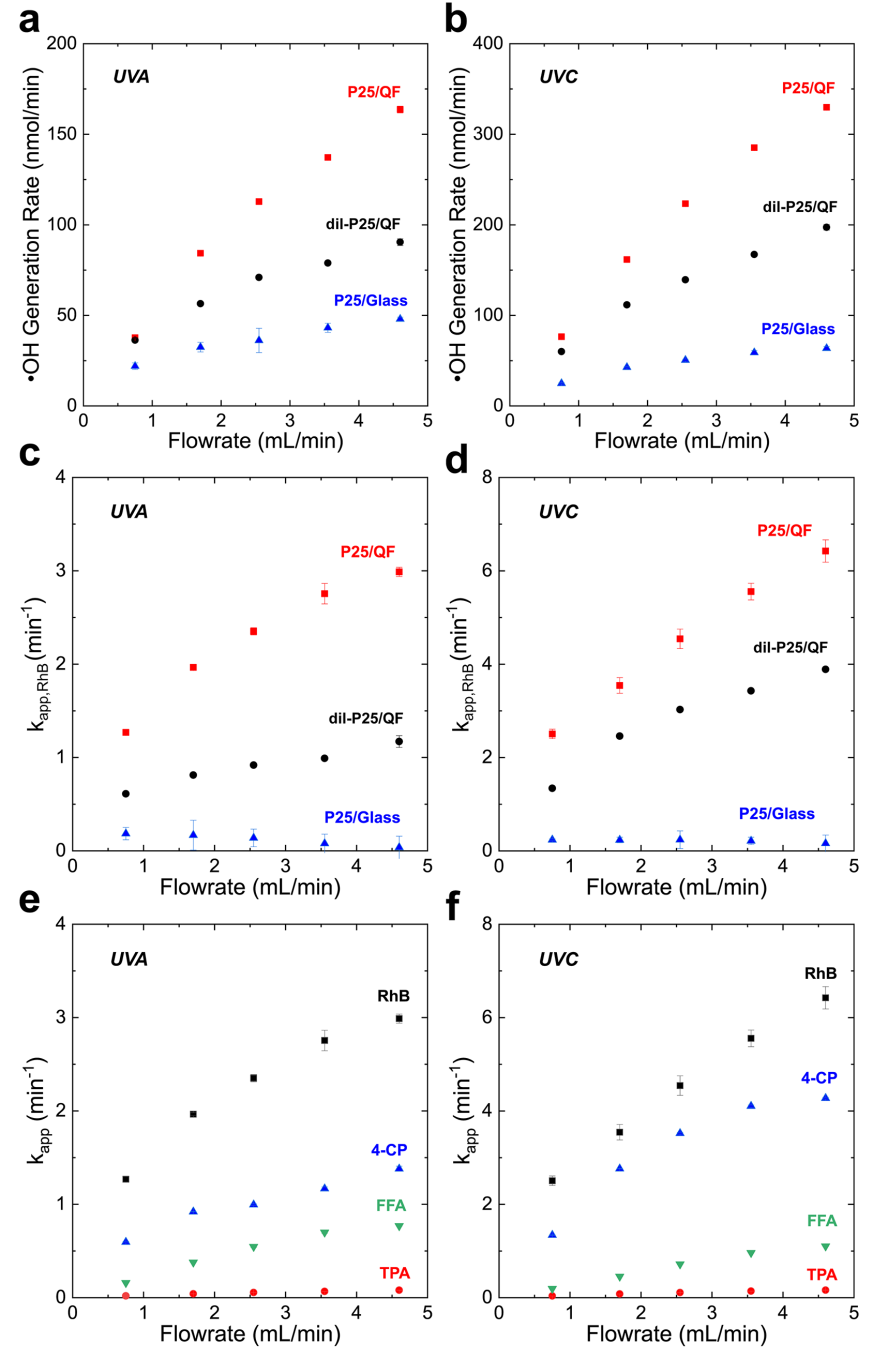
Performance of P25/QF, dil-P25/QF and P25/glass photocatalyst under UVA and UVC illumination, as compared by (a, b) ·OH generation rate, (c, d) RhB apparent degradation rate constants, and (e, f) apparent degradation rate constants for RhB, 4-CP, FFA, and TPA using P25/QF. All graphs contain error bars from triplicates of tests.

We characterized the bulk and surface properties of P25/QF samples, with relevant parameters and calculations provided in Table S1 and Discussion S1 in the Supporting Information, respectively. We compared the performance of our P25/QF photocatalyst to two other systems, dil-P25/QF and P25/glass. The P25/QF photocatalyst contains an ∼0.65 μm thick mesoporous titania coating with a BET surface area of 8.22 m^2^/g on the macroporous (ϕ ≈ 0.95) QF support and a catalyst mass loading of ∼14% w/w. The dil-P25/QF photocatalyst has 6.5× less surface area at 1.26 m^2^/g. The BET adsorption/desorption isotherms for P25/QF and dil-P25/QF are listed in Figure S6. The P25/glass photocatalyst surface area could not be measured by BET analysis due to size limitations in the BET measuring equipment. However, we postulate that the surface area is very low, which is strongly supported by the ·OH generation and RhB degradation data we gathered using P25/glass, as shown in Figures 2a–d. The P25/QF photocatalyst attained a surface area-to-reactor volume ratio over 940,000 m^2^/m^3^ ± 6%, a value much larger than photocatalysts reported in many other works, whether immobilized or in suspension.^7^ A detailed explanation of the surface area-to-reactor volume ratio calculation is included in the Supporting Information.

### 3.2 Multiactivity Test of Immobilized Photocatalysts in the MFR

To accurately judge the performance of our supported photocatalytic system, we performed a multiactivity assessment using four common probe molecules from different chemical classes as detailed in Table 1. Supported photocatalysts were exposed to UV irradiation in a single pass through the MFR, and the relevant concentrations of reactants were measured at the inlet and outlet of the MFR. To minimize the effects of differences between synthesized photocatalysts, we used the same photocatalyst for as many tests as possible; for example, the same photocatalyst was used for all 12 of the RhB and TPA degradation vs flow rate tests under UVA and UVC. The run-to-run reproducibility of these tests, as indicated by the low standard deviation in our triplicate tests, is a testament to the stability of our P25/QF photocatalyst system.

**Table 1:** E_EO_ (kWh/m^3^) Values for the Degradation of RhB, 4-CP, FFA, and TPA by P25/QF (with UVA and UVC) and a Traditional UVC/H_2_O_2_ AOP^a^

**Probe Molecule**	**Chemical Class**	**UVA P25/QF**	**UVC P25/QF**	**UVC/H**_**2**_**O**_**2**_**AOP**
RhB	Dye	0.90	0.60	4.57
4-CP	Aromatic/Chlorohydrocarbon	1.95	0.90	3.47
FFA	Alcohol	3.50	3.48	183
TPA	Organic Acid	33.8	23.7	63.2

a A lower E_EO_ indicates a more energy efficient degradation process.

Initially we tested the ·OH production rate from the three photocatalytic systems. ·OH groups are the primary oxidizers in most AOPs given their high oxidation potential of 2.8 V,^41^ and therefore it is important to determine the ·OH generation rate in the MFR for the three photocatalytic systems. Transformation of TPA to hTPA, observed via fluorescence, served as an indicator for ·OH.^34,35^ Pseudo-steady-state ·OH generation rates were estimated based on the following assumptions: (1) TPA reacts selectively with free ·OH, (2) the excess concentration (500 μM) of TPA in the water reacts with bulk ·OH, and (3) the reaction yield of ·OH with TPA to form hTPA is ∼30%.^35^ P25/glass, dil-P25/QF, and P25/QF samples were tested using varying flow rates (0.75 to 4.6 mL/min) and under UVA or UVC sources. In both UV regimes, the trend in the effective ·OH generation rate followed the trend in photocatalyst surface area with P25/QF > dil-P25/QF > P25/glass (Figure 2a, b). Furthermore, the observed ·OH generation rates improved with flow rate for all three systems, as measured by the TPA reaction. This relation between flow rate and performance suggests that the reactions are mass transfer limited.^42^

The breakdown of the triphenylmethane dye, RhB, is important in numerous industrial wastewaters (e.g., textile, printing, food, and cosmetics), and the capacity to degrade such pollutants acts to confirm the ability for a given technology to destroy micropollutants in potable water applications. The dye exhibits pseudo-first-order kinetics and, given its popularity in similar studies, serves as a metric for comparison.^37,43^ A simple kinetic model for the photodegradation of RhB, and the other probe molecules in Table 1,^44,45^ is proposed as
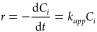
3
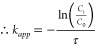
4where the rate of degradation of the probe molecule, *r*, is described by an apparent rate constant, *k*_*app*_ [min^–1^], and the concentration of the probe molecule, *C*_*i*_, as well as the residence time in reactor, τ. Here, *k*_*app*_ accounts for the rate constants of several factors, including the internal mass transfer, external mass transfer, illumination intensity, and applicable reaction rate constants (i.e., the probe molecule with ·OH, photoholes, and any other reactive oxygen species (ROS)).

Photodegradation of RhB, 4-CP, FFA, and TPA solutions were measured for the different photocatalyst platforms under controlled UV fluence and flow rate with a single pass through the MFR. The apparent degradation rate constant, *k*_*app,RhB*_, again follows the trend in photocatalyst surface area with P25/QF > dil-P25/QF > P25/glass (Figure 2c, d).

The P25/QF system achieved higher observed ·OH generation rates and RhB photodegradation rate constants under UVC illumination compared with UVA irradiation. This difference was likely caused by a combination of factors, including the increase in light absorbance from UVA to UVC wavelengths (Figure S7) and the photolysis of photogenerated H_2_O_2_ by UVC into ·OH. The ability of QF to transmit UVC radiation makes the QF support highly appealing for integration within current AOP systems.

While RhB is widely used as a probe molecule for photocatalytic studies, accurate characterization of the activity of a photocatalyst requires testing against multiple probes from various chemical classes, as detailed in Table 1.^46^ Therefore, we tested the ability of our P25/QF system to degrade 4-CP, FFA and TPA probe molecules. Figures 2e and f shows the apparent degradation rate constants *k*_*app*_ for all four of our probe molecules under UVA and UVC excitations, respectively, using P25/QF. All probe molecules illustrate mass transfer limited behavior indicated by higher flow rates resulting in faster *k*_*app*_. The trend in *k*_*app*_ for the P25/QF system is RhB > 4-CP > FFA > TPA. While a full mechanistic understanding of this trend is beyond the scope of this work, it is important to note that the operating pH of 6.5 for these studies is at or near the point of zero charge of P25.^46,47^ Therefore, we do not expect strong electrostatically driven adsorption between our probe molecules and the P25 surface. Furthermore, higher flow rates, i.e., reduced time for adsorption, led to an increased degradation rate for all of the probe molecules. This observation suggests that mass transfer effects, not kinetically limited adsorption, play a dominant role in the system performance. Lastly, the ·OH generation rates for the P25/QF system are remarkably high with steady state ·OH concentrations in the MFR estimated to exceed 72 μM at a flow rate of 4.6 mL/min based on our TPA measurements. We posit that these high ·OH concentrations play a significant role in the degradation of the probe molecules, but we cannot rule out the role of photoholes driving the degradation of the adsorbed species.

Using eq 2, we calculated the E_EO_ for each sample and UV regime (Table 1). The E_EO_ values for each probe molecule followed the trend P25/QF UVC < P25/QF UVA < UVC/H_2_O_2_. We observed a 662%, 286%, 5159%, and 167% improvement in the E_EO_ values between the P25/QF UVC and UVC/H_2_O_2_ AOP cases for RhB, 4-CP, FFA, and TPA, respectively. Furthermore, our E_EO_ for RhB of 0.6 kWh/m^3^ for P25/QF under UVC is a substantial improvement over the values found for many TiO_2_/UV systems in literature.^11,21,37,39^

### 3.3 Catalyst Loading Effects

**Figure 3:**
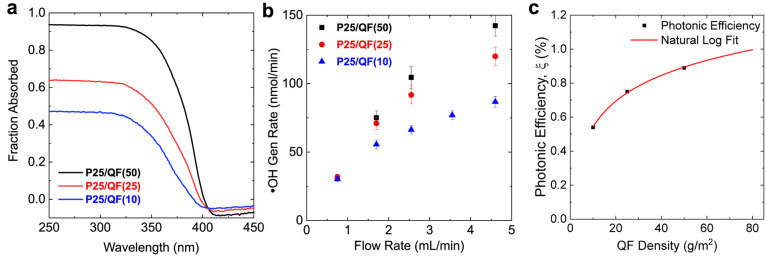
Evaluating the effect of QF density on the photocatalytic performance of P25/QF(ρ) in single-pass flow tests: (a) absorptance spectra, (b) ·OH generation rate under UVA excitation, and (c) calculated photonic efficiency metrics for P25/QF samples with varying QF density (ρ) and extrapolated values using a natural log fit (solid line).

Once we established the benefits of our P25/QF system compared with UVC/H_2_O_2_ for ·OH generation and pollutant degradation, we further explored the optical and mass transport impacts of the P25/QF sample by adjusting the catalyst loading. We implemented different densities of the QF support loaded with the same thickness of porous P25 on a per fiber basis. We denote these QF supports as QF(ρ) where ρ represents the area density of the QF in units of grams of QF per m^2^ of QF surface area. Increasing ρ increases the overall catalyst loading and P25 surface area in the MFR. We measured the optical absorption of the P25/QF(ρ) samples (Figure 3a) using an integrating sphere with the sample center mounted (see the Experimental Information section of the Supporting Information) and observed an increase in the fraction of UV absorbed with increasing ρ. To further characterize the optical properties of the photocatalyst, the absorption of P25/QF samples with increasing thicknesses of P25 was used to calculate the attenuation coefficients at 254 and 365 nm (see Discussion S1 and Figure S8 for details).

To better understand the effect of UV absorption, catalyst surface area, and flow rate on the apparent reaction rate, we investigated the ·OH generation and RhB degradation rates (Figure 3b and Figure S9, respectively) as a function of flow rate and ρ; see Figure 3b. At low flow rates (e.g., <2 mL/min) increasing the catalyst surface area with higher ρ QF supports has a negligible effect on the apparent reaction rate of the system. We posit that this is due to the system being mass transfer limited under these low flow conditions (e.g., *Re*_i_ ≈ 0.17). Note that *Re*_i_ is the modified Reynold’s number for flow-through porous media (see eq S7). Above 2 mL/min the mass transfer limitations are relaxed, and we observe a significant increase in the reaction rate with a higher catalyst loading due to increased optical absorption. Furthermore, as the flow rate is increased, the difference between the reaction rates for a given ρ also increases, which we attribute to improved mass transfer. We posit that QF supports with higher ρ yield more microscale turbulence, thus further increasing the apparent reaction rate.

Optimization of the decontamination system for overall energy efficiency requires normalizing to the total photons incident in the system, which is represented by the photonic efficiency (eq 5).^48^
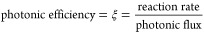
5We plot the photonic efficiency (ξ) in Figure 3c for ·OH generation under UVA excitation as a function of ρ. All ξ values were calculated at the maximum flow rate of 4.6 mL/min to mitigate mass transfer limitations on the apparent reaction rate. In general, higher density QF supports increase ξ but with diminishing improvements.^49^ ξ is ∼0.9% for the QF(50) support. The relationship between ξ and ρ can be simplified to a relationship between the reaction rate and photocatalyst surface area. This simplification arises from the fact that the photonic flux (i.e., denominator of ξ) is held constant by the light source. Furthermore, the surface area of the catalyst is linearly proportional to the mass of the catalyst, which is directly related to ρ. So, this raises the question, what is the expected relationship between the photocatalytic reaction rate and photocatalyst surface area? In general, increasing the surface area will increase the number of active sites for the reaction.^50^ Thus, the reaction rate is expected to increase but does not always due to complications with synthesizing photocatalysts with a consistent density of active sites. When an increasing trend in reactivity with photocatalytic surface area has been observed in the literature, it typically extends to some saturation point at which the reaction rate plateaus with increasing surface area. Bloh attributes the plateau in the reaction rate at high surface areas to the system being photon-limited, i.e., there are not enough photons to reach the additional active sites.^50^Figure 3c shows a similar trend, with the ξ increasing with ρ, i.e., increased surface area, but with diminishing improvements. A natural log function provides an excellent fit (*R*^2^ = 0.9988) to the ξ vs ρ data points and predicts negligible improvement in the ξ above 80 g/m^2^ QF density. This agrees with the absorptance data in Figure 3a, which report that ∼80% of the UVA light is already absorbed in the P25/QF(50) system. Thus, we posit that a QF support with a ρ of 80 g/m^2^ will yield a maximum ξ of 1% with further increases in ρ being photon-limited.^7,12,37,51,52^

### 3.4 Impact of Irradiance and Flow Rate

**Figure 4:**
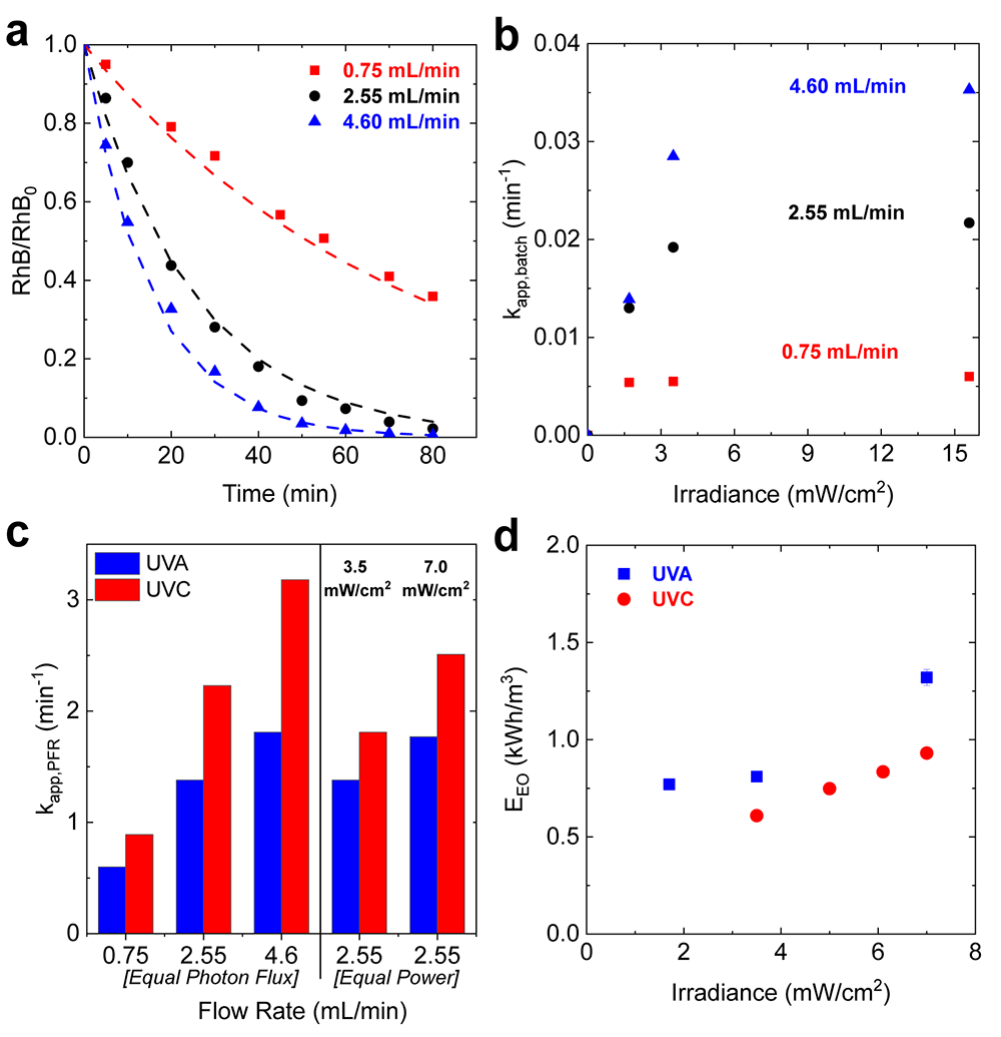
Effect of flow rate and illumination intensity on RhB degradation in recycled batch tests. (a) RhB degradation under 3.5 mW/cm^2^ UVA, with batch data fit to a single-pass conversion model (dashed lines). (b) Apparent rate constant for RhB degradation under different UVA intensities and flow rates. (c) Rate constants for single-pass degradation of RhB under UVA and UVC controlled for photon flux and lamp output power. (d) E_EO_ for RhB degradation by P25/QF(50) under UVA and UVC with a 2.55 mL/min flow rate.

To better understand the P25/QF photocatalyst performance with respect to UV intensity and longer-term performance, we implemented recycled batch tests. These tests were used to measure pollutant degradation trends and calculate apparent reaction rates. Degradation tests were conducted on 10 μM RhB solutions in DI water under different recycled flow rates between 0.75 to 4.6 mL/min (Figure 4a). UVA irradiation was used via a UV lamp, allowing for 30 min of warming time for the lamp. Samples were taken from both the batch reactor and the single-pass effluent to estimate the single-pass degradation of RhB in the UV/catalyst system. The model in eq 6 was used to compare the values:^53^
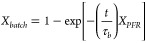
6with *X*_*batch*_ denoting the conversion (i.e., degradation) of RhB measured from the batch and *X*_*PFR*_ denoting that from the PFR reactor sample, *t* being the time passed, and τ_*b*_ denoting the average residence time of species in the batch reactor (i.e., *Vol*_*batch*_/*F*). The single-pass conversion data was used to model the degradation curves in Figure 4a (dashed lines) and provide a good fit with the measured batch data (*R*^2^ > 0.99 in all cases). A least means squared analysis of the experimental vs calculated residuals was implemented to extract apparent rate constants with greater statistical significance.

We tested the performance of P25/QF under various flows and UV irradiances to better understand the optical conditions. We regulated the illumination power between 1.5 to 15.3 mW/cm^2^ by adjusting the lamp distance from the photocatalytic system and measured the resultant degradation of RhB at different flow rates (Figure 4b). At low flow rates, the degradation rate of RhB reached a maximum at lower light intensities. However, increasing the illumination yields higher rates of photodegradation at higher flows. This observation supports the previous hypothesis that the flow rate is a primary limiting factor in the current system and that the system could improve with scaling-up to more applicable, higher flow conditions.

We additionally tested the degradation of RhB under different UV illumination sources, UVA and UVC, at equivalent power and photon flux. In both cases, UVC irradiance resulted in faster degradation of RhB than UVA (Figure 4c). While this is generally unsurprising given the additional absorption of UVC compared with UVA for the P25 catalyst, most supported systems suffer from the inability to transmit UVC to additional photoactive sites within a given volume. The QF support exhibits negligible parasitic absorption in the UVC, allowing for deeper penetration of UVC light into the QF support and the use of standard germicidal light sources. This advantage was further quantified through the recycled-batch E_EO_ calculations for RhB degradation (Figure 4d), which not only showed that UVC performs better energetically than UVA with P25/QF photocatalysts but indicated that lower lamp power yielded better E_EO_ metrics. Thus lower light intensity per volume of photocatalyst could be used to optimize photocatalyst performance.

### 3.5 Water Source Impacts on P25/QF Activity

Most catalysts suffer from the undesired fouling of active sites over time. Initially, we tested the 24 h stability of P25/QF for the degradation of a continuous 10 μM RhB feed in DI water as an indicator of reactant fouling (Figure S10). Across the test, we observed a <5% variance in RhB degradation.

We also tested the impacts of alkalinity, conductivity, and organic load on our P25/QF system by selectively adding chemical components typically found within surface waters.^3,26,54^ First, we added increasing amounts of sodium bicarbonate to DI water in order to provide alkalinity in the range found in soft to medium-hard drinking water while maintaining the pH at 6.5. Carbonate and bicarbonate are often implicated as primary scavengers of ·OH within drinking water systems, with second-order reaction rate constants of 3.9 × 10^8^ and 8.5 × 10^6^ (L mol^–1^ s^–1^), respectively.^26,55^ An addition of up to 800 mg/L of NaHCO_3_ (i.e., 400 ppm alkalinity as CaCO_3_) had a negligible impact on the photodegradation of RhB with the P25/QF photocatalyst under UVA or UVC illumination. We further added 70 mg/L CaCl_2_ to augment the electrical conductivity. This also did not alter the performance of the photocatalyst. We then added 2 mg/L of unfiltered humic acids (HA) and refer to our mixture of bicarbonate, calcium chloride, and HA as a SSW, with water quality parameters provided in Table 2.

**Table 2:** Synthetic Source Water (SSW) and Mississippi River Water (MRW) Characteristics

**Parameter**	**SSW**	**MRW**
pH	6.5	7.8
Temperature (°C)	20	20
UVT_365_ (%)	93.6	-
UVT_254_ (%)	69.2	78.4
Humic Acids (mg/L)	2.0	-
TOC (mg-C/L)	5.0	1.9
Alkalinity (as mg CaCO_3_/L)	200	-

**Figure 5:**
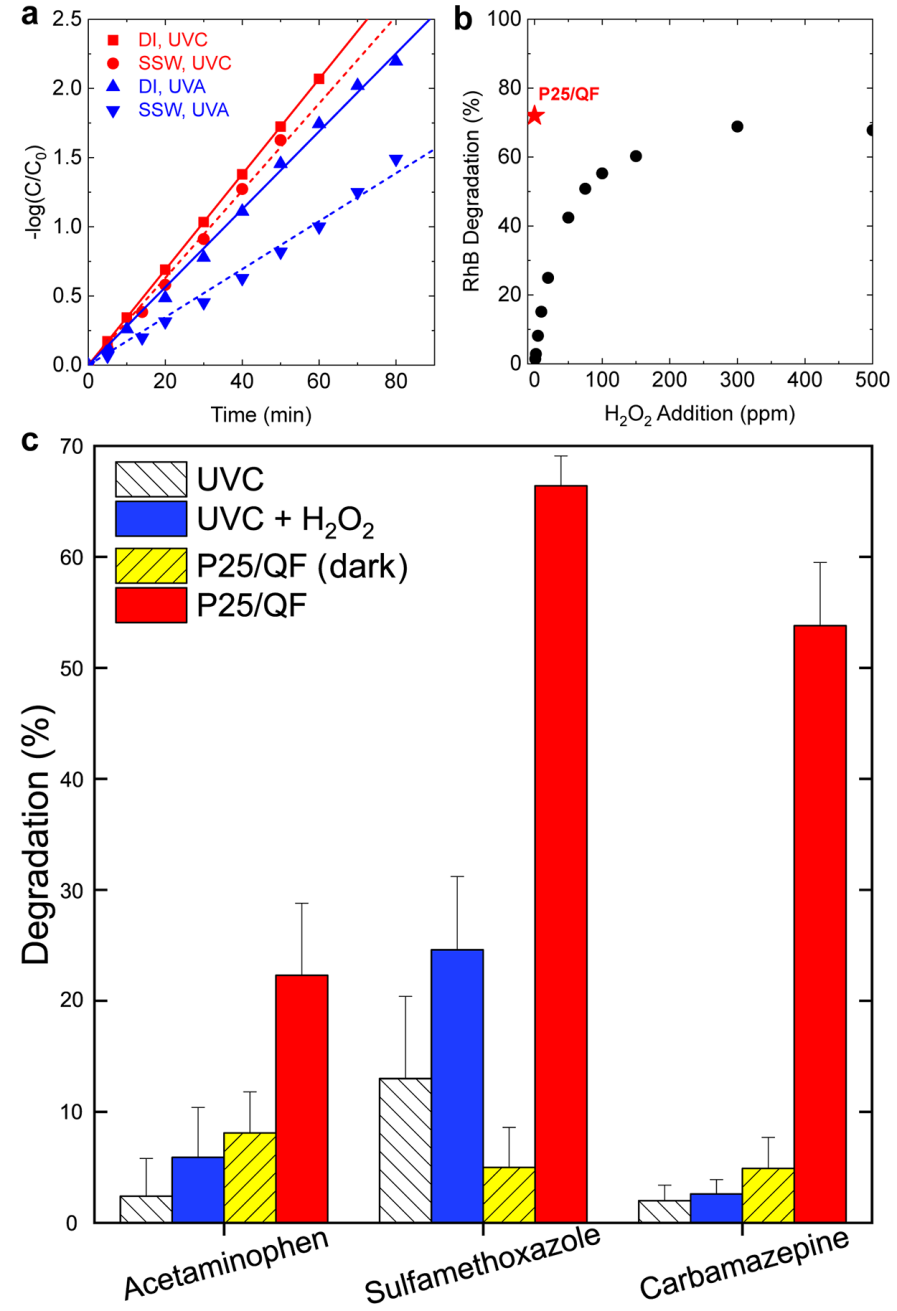
Performance of P25/QF in various water sources. (a) First-order kinetic breakdown of RhB by P25/QF under UVA and UVC light in both DI and SSW. (b) Comparative RhB degradation in SSW using P25/QF under UVC illumination and a traditional H_2_O_2_/UVC AOP with various H_2_O_2_ concentrations. (c) Degradation of pharmaceutical compounds in MRW using various AOPs and a dark control for P25/QF.

The unfiltered HA had an adverse effect on the P25/QF performance due to adsorption of HA to the P25 surface (i.e., catalyst fouling), as shown in Figure 5a. The HA may also reduce UV transmittance and scavenge ROS.^56−58^ Notably, the impact of HA on the P25/QF performance was greater when under UVA illumination than under UVC. In addition, we conducted a 24 h test of RhB degradation with P25/QF in SSW to examine ongoing fouling impacts of HA. As shown in Figure S10, the activity of P25/QF decreased by ∼60% over 24 h with a 2 mg/L HA addition at near-neutral pH. This suggests that a prefiltration step for the P25/QF system could prove useful for system longevity. Alternatively, there may be value in backwashing the photocatalyst periodically in applied systems to help reduce the buildup of contaminants on the active surface. Both filtration and associated backwashing are common treatment practices upstream of traditional UVC/H_2_O_2_ processes.

To compare the performance of our P25/QF photocatalyst to that of a traditional H_2_O_2_/UVC AOP in SSW, we measured the degradation of RhB in both systems. For the H_2_O_2_/UVC test, we implemented concentrations of 5–500 ppm of H_2_O_2_ and measured the relative RhB degradation under UVC illumination. In many commercial H_2_O_2_/UVC reactors, 50 ppm represents a maximum H_2_O_2_ dosage utilized, with concentrations of 5–20 ppm of H_2_O_2_ more commonly used, depending on the specific treatment goals.^6^ Increasing the H_2_O_2_ concentration resulted in better RhB degradation (Figure 5b) with diminishing returns for increasing the H_2_O_2_ doses above 50 ppm. In the absence of light, no RhB degradation was observed. Comparatively, the P25/QF photocatalyst under UVC illumination attained an RhB degradation 2.5× greater than that through the addition of 50 ppm of H_2_O_2_, at a controlled flow rate of 4.6 mL/min. Even with a dosage of 300 ppm of H_2_O_2_, which provided the maximum RhB degradation in the MFR, the P25/QF exhibited superior pollutant degradation. This observation is a very promising result indicative of the potential benefits of P25/QF for a chemical-free AOP in water treatment.

### 3.6 Degradation of Pharmaceutical Micropollutants in Mississippi River Water

The P25/QF photocatalyst was also tested against various pharmaceutical compounds (i.e., micropollutants) of concern. MRW was spiked with 10 ppm of pharmaceuticals (acetaminophen, sulfamethoxazole, and carbamazepine) and introduced to the MFR at 4.6 mL/min under various catalytic conditions. The water quality profile for the MRW is provided in Table 2, and the results of the pharmaceutical degradation test are listed in Figure 5c. Note the reported pharmaceutical degradation efficiencies in Figure 5c are for a residence time in the MFR of less than 30 s. This timeframe is very short compared to other photocatalytic pharmaceutical degradation studies which employ residence times up to 2 h.^59−61^ The P25/QF under UVC illumination greatly outperformed the UVC alone case and UVC with 6 ppm of H_2_O_2_ addition for all three pharmaceuticals. While sulfamethoxazole underwent the greatest degradation under UVC (13%), H_2_O_2_/UVC (24.6%), and P25/QF (66.4%), the greatest improvement in the P25/QF activity over H_2_O_2_/UVC AOP was for carbamazepine. The comparative E_EO_ for pharmaceutical breakdown using these technologies is provided in Table 3. The P25/QF system in MRW demonstrates an ∼300% improvement in the degradation of acetaminophen and sulfamethoxazole and >2800% improvement for carbamazepine vs a traditional H_2_O_2_/UVC AOP. Furthermore, testing with P25/QF under dark conditions suggested limited adsorption of each compound on the P25 surface. The long-term impact of this adsorption requires further investigation.

**Table 3:** Comparison of E_EO_ for Pharmaceutical Compound Degradation Using Traditional H_2_O_2_/UVC and Our P25/QF Photocatalyst

**E**_**EO**_ **(kWh/m**^**3**^**)**	**Acetaminophen**	**Sulfamethoxazole**	**Carbamazepine**
H_2_O_2_/UVC	17.25	3.69	40.04
P25/QF	4.07	0.96	1.35
Improvement	324%	284%	2866%

## 4 Conclusion

We developed an immobilized, titania-based photocatalyst and demonstrated its ability to degrade a variety of organic compounds in different aqueous environments with efficiencies exceeding commercial H_2_O_2_/UVC AOPs by over 2800%, as measured through the E_EO_ metric. Our P25/QF photocatalytic AOP is unique in that it implements dual-scale porosity—that is, a mesoporous catalyst film affixed to a macroporous catalyst support structure—to overcome limitations in mass and optical transport that currently plague the field of photocatalytic advanced oxidation. We analyzed the fundamental improvements of the P25/QF system compared to other photocatalytic architectures and found that, while the current system provides substantial benefits to photocatalytic activity, further tuning of the photocatalyst density, incident optical power, and flow rate could improve the performance further, in terms of both photonic efficiency and overall degradation rates. We also confirmed the activity of the P25/QF photocatalyst under both UVA and UVC illumination, opening the door to its use in a wide range of technological platforms. Uniquely, our QF scaffold transmits both light sources with >90% efficiency and is thus better positioned for near-term application with UVC sources than polymer, metallic, or even glass support systems.^11,21−25^ While the potential to use UVA and solar radiation is energetically appealing, we believe that serious investigations of photocatalysts for large-scale water treatment applications are bolstered by examining their efficacy under UVC radiation that is already implemented in many commercial systems. Further optimization of the P25/QF system could benefit tertiary water treatment efforts by eliminating the need for chemical additives, reducing the energetic cost of treatment and simplifying the equipment used in treatment trains to remove harmful organic compounds.

## Supporting Information Available

Additional characterization data of the photocatalyst, quartz fiber support, and transport modeling details (PDF)
