# Supplementary material for: Efficient Chemical-Free Degradation of Waterborne Micropollutants with an Immobilized Dual-Porous TiO2 Photocatalyst
Source: ACS ES T Eng. 2023 Aug 3;3(11):1694–705. doi: 10.1021/acsestengg.3c00191 (PMC10644339; doi:10.1021/acsestengg.3c00191)
Supplement: Supplementary file 1 — ee3c00191_si_001.pdf [file ee3c00191_si_001.pdf]

Supporting Information for

Efficient Chemical-Free Degradation of Waterborne Micropollutants with an Immobilized Dual-Porous TiO_2 Photocatalyst

Daniel E. Willis[†], Ella C. Sheets[†], Mary R. Worbington[†], Madhusudan Kamat[§], Sarah K. Glass[†], MaCayla J. Caso[†], Tochukwu Ofoegbuna[†], Liz M. Diaz[†], Caleb Osei-Appau[§], Samuel D. Snow[§], Kevin M. McPeak[†]

† Gordon and Mary Cain Department of Chemical Engineering, Louisiana State University, Baton Rouge, Louisiana 70803, USA

§ Department of Civil and Environmental Engineering, Louisiana State University, Baton Rouge, Louisiana 70803, USA

Experimental Information: Photocatalyst Characterization

X-ray Diffraction (XRD): The crystal structure of our P25/QF films was identified through out-of-plane XRD using a PANalytical X-ray diffractometer operating at 45 kV and 40 mA. The θ-2θ radial scan was performed over the range 30-45º with a step size of 0.01º and dwell time of 120 s, using Cu Kα (λ=1.54 Å) as the radiation source.

UV-Vis Spectroscopy: The optical absorbance of QF films without P25 pre- and post-binder removal was measured in a Lambda 900 spectrophotometer with an integrating sphere. We initially collected a baseline spectrum with the integrating sphere empty. Then spectra were collected from a 25 mm x 25 mm piece of QF before and after it was heated in ambient air. The QF was center mounted in the integrating sphere with a wavelength range of 250 – 450 nm.

Scanning Electron Microscopy (SEM): A Hitachi S4500 scanning electron microscope was used for all electron micrographs. We did not coat the samples with metal or carbon prior to imaging.

X-ray tomography: A QF sample was scanned at the University of Texas High-Resolution X-ray CT Facility on a Zeiss Xradia 620 Versa. The 4X detector objective was employed, unbinned, with the X-ray source, distanced 10.0 mm, and the detector distanced 23.9 mm from the center of the stage, resulting in 1.00-micron resolution. The X-ray source was set to 80 kV and 10 W, and no X-ray prefilter was used. A total of 2401 projections were acquired over ±180 degrees of rotation. The resulting volume was reconstructed as a series of 16bit TIFF images with a center shift of -4.5, beam-hardening correction of 1, rotation of 8 degrees, and byte scaling endpoints of -0.02 and 0.22.

Brunauer–Emmett–Teller (BET): BET analysis was performed using a Micromeritics AutoChem II. The photocatalyst (P25/QF) was added to a glass cell. The sample was then degassed using nitrogen gas and reweighed. Nitrogen was adsorbed onto the sample by using liquid nitrogen to lower the temperature. By measuring the quantity of nitrogen adsorbed at specific pressures, the BET surface area was calculated.

In the BET analysis, it was determined that the surface area of the QF support was negligible compared to that of the porous P25 coating, indicating that the specific surface area of P25 will be greater than the BET surface area of P25/QF. The area of QF was calculated by assuming the QF consists of idealized quartz cylinder rods of 6.2 μm diameter with a material density of 2.65 g/cm^3. We took the total mass of QF weighed prior to coating with P25, determined the cylindrical volume, then calculated a total length of QF rods. We then used this length to determine QF surface area, which was <1% of the area measured by BET for P25/QF samples. Given this, we then extrapolated the weight fraction of P25 within the P25/QF sample (~22%). To provide a metric for comparing our P25/QF system to other photocatalytic systems, we used the BET

surface area (8.22 m^2/g), an average weight of 2x2" squares of P25/QF (0.144 g), and the reactor volume (1.25 cm^3) to calculate a surface area to reactor volume ratio of 940,000 m^2/m^3 ± 6%.

Supplemental Figures

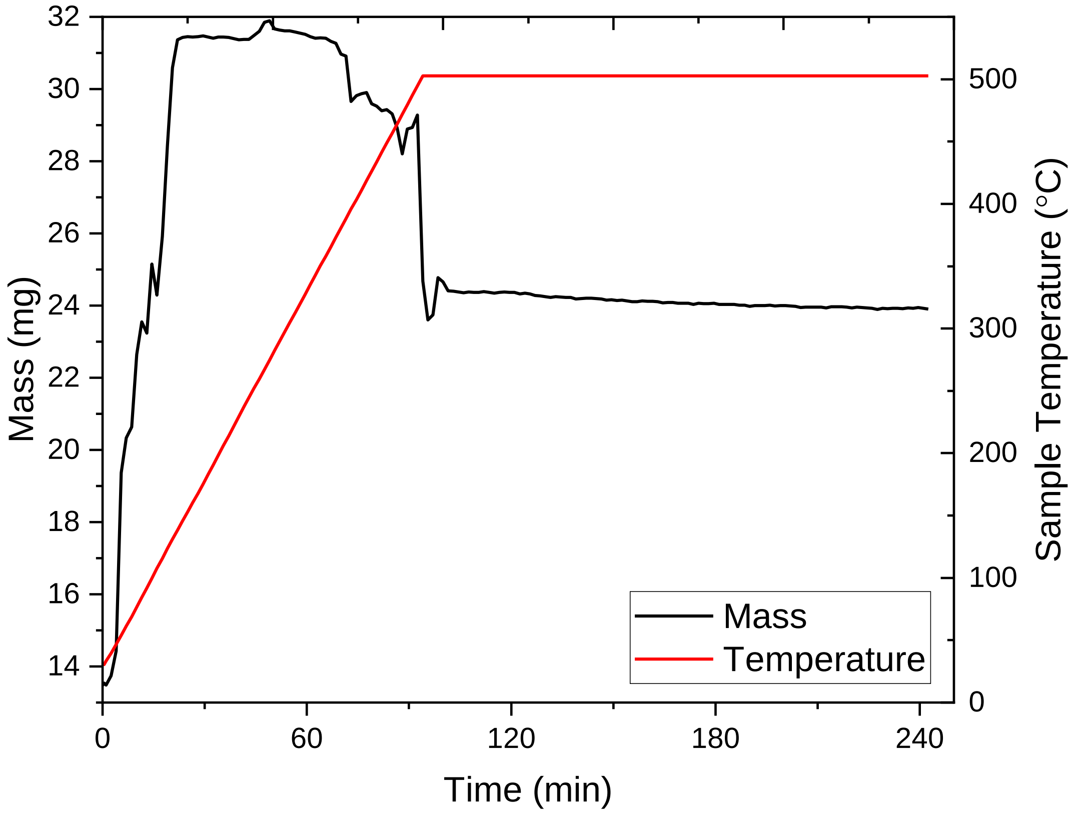

Figure S1. Thermogravimetric analysis (TGA) scan of quartz veil with an epoxy binder in air showing degradation of the binder at 500°C.

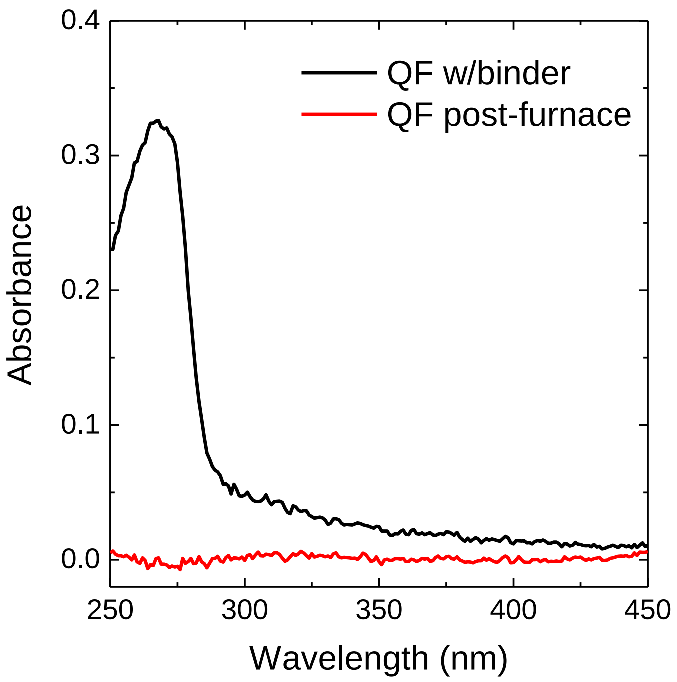

Figure S2. UV absorbance of QF substrates before (black) and after (red) epoxy binder removal through heating in an ambient furnace at 500°C for 2 h.

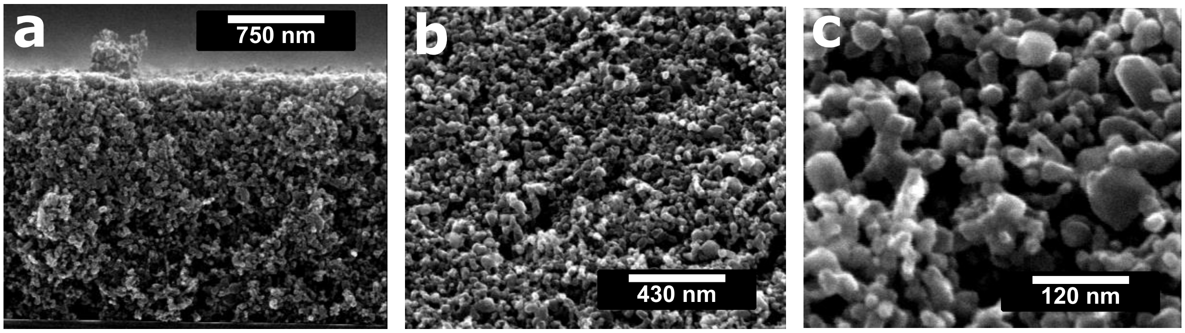

Figure S3. (a) Side-view SEM micrographs of the porous P25 film. (b) The P25 film surface viewed at a 45-degree tilt and (c) at higher magnification.

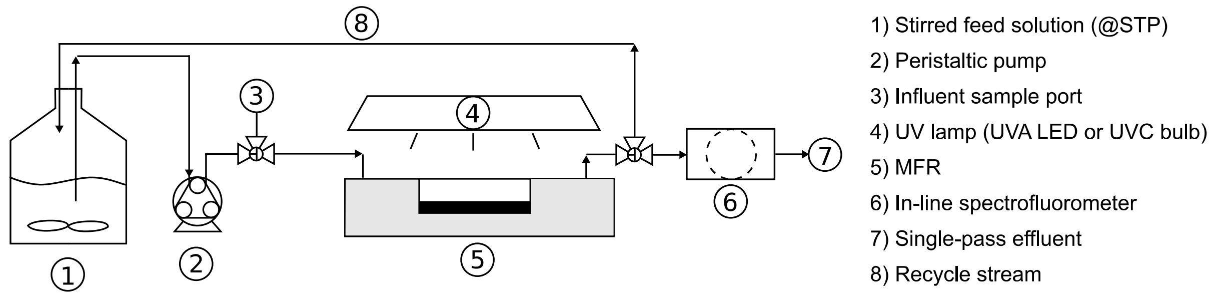

Figure S4. Schematic of the continuous-flow reaction system with both single-pass flow measurements (analyzed with an in-line spectrofluorometer) and/or a recycled stream for recycled batch testing.

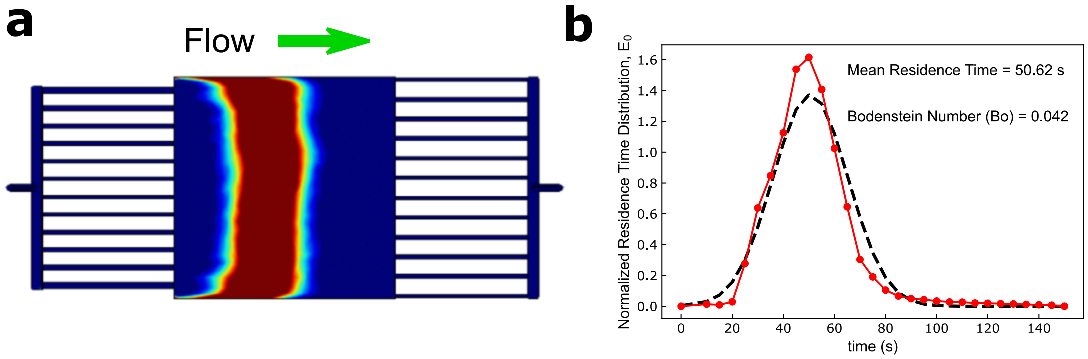

Figure S5. (a) A simulated pulse flow in the milliflow reactor (MFR). (b) Measured residence time distribution and the dispersion function based on the calculated Bodenstein Number (Bo) for this system.

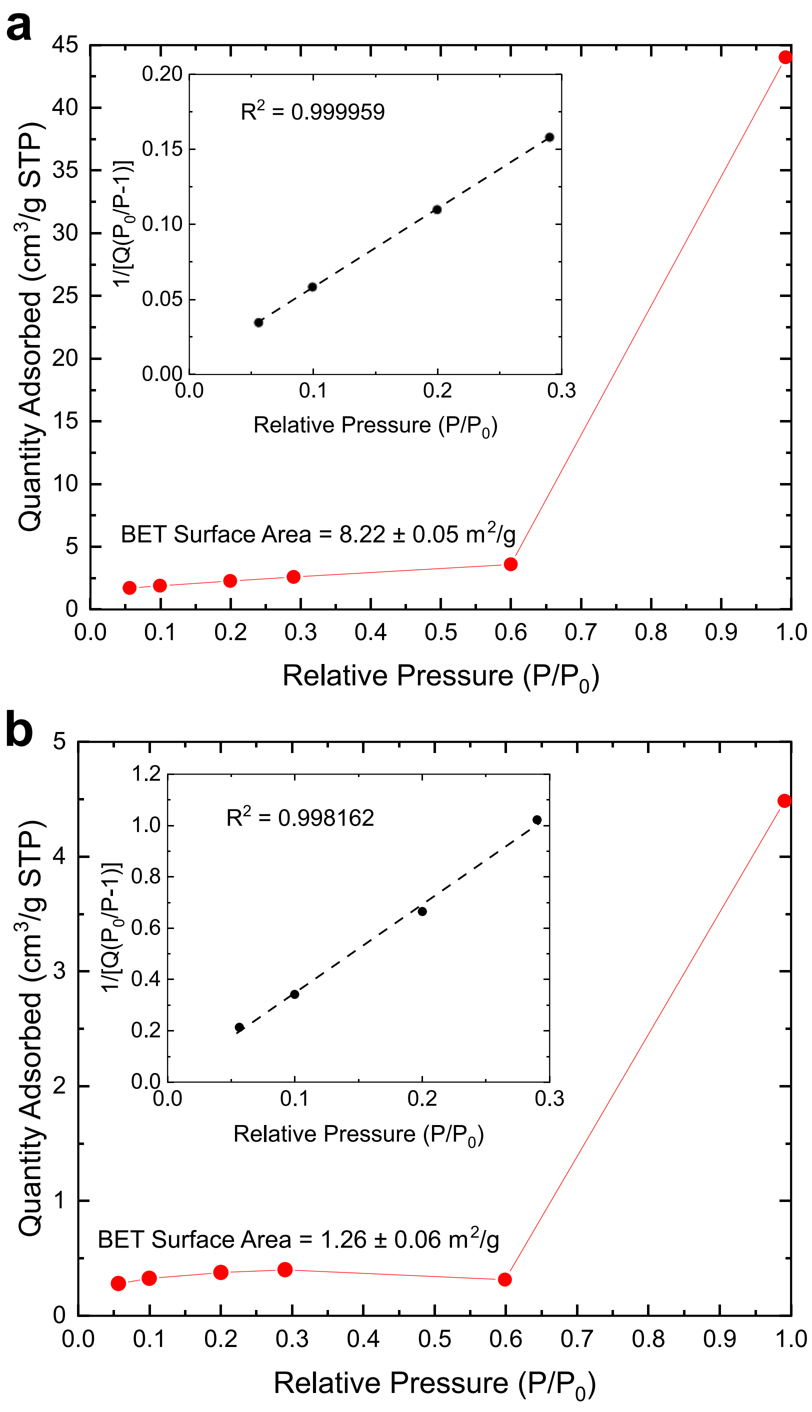

Figure S6. (a) The BET surface area of P25/QF was calculated through a nitrogen adsorption-desorption isotherm with an insert of the fit BET plot. (b) The BET surface area of dil-P25/QF with an insert of the fit BET plot.

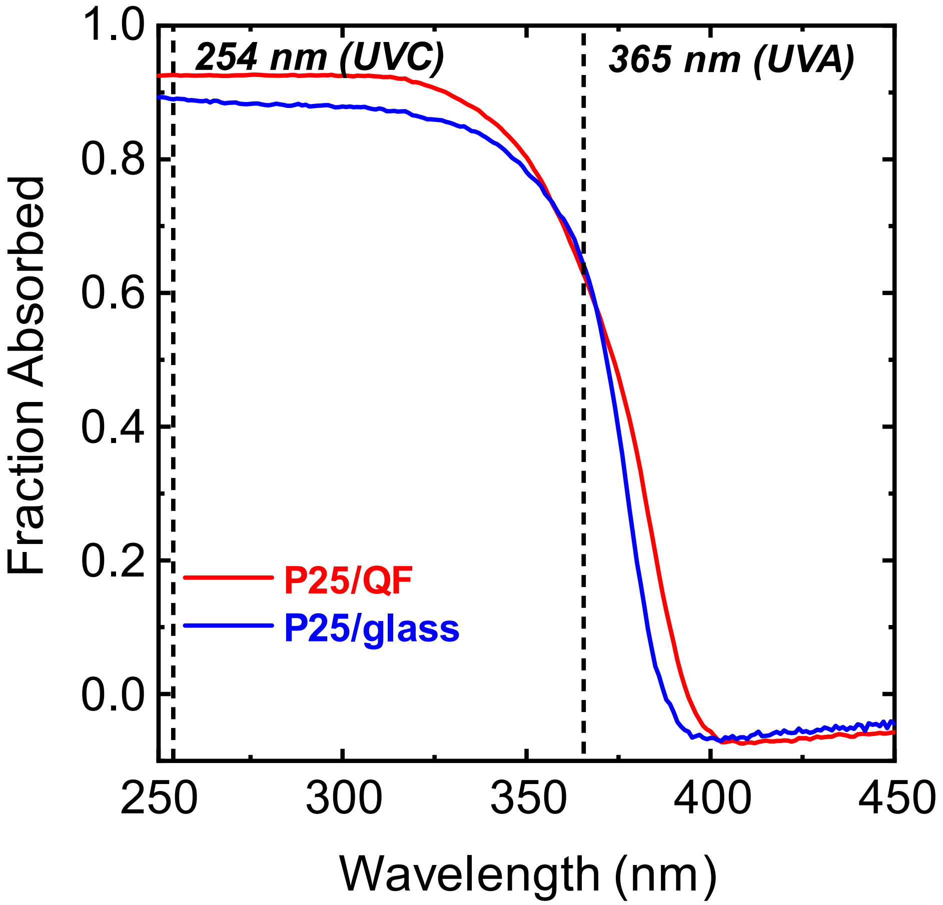

Figure S7. Comparison of fractional absorption of UV light for P25/QF and P25/glass.

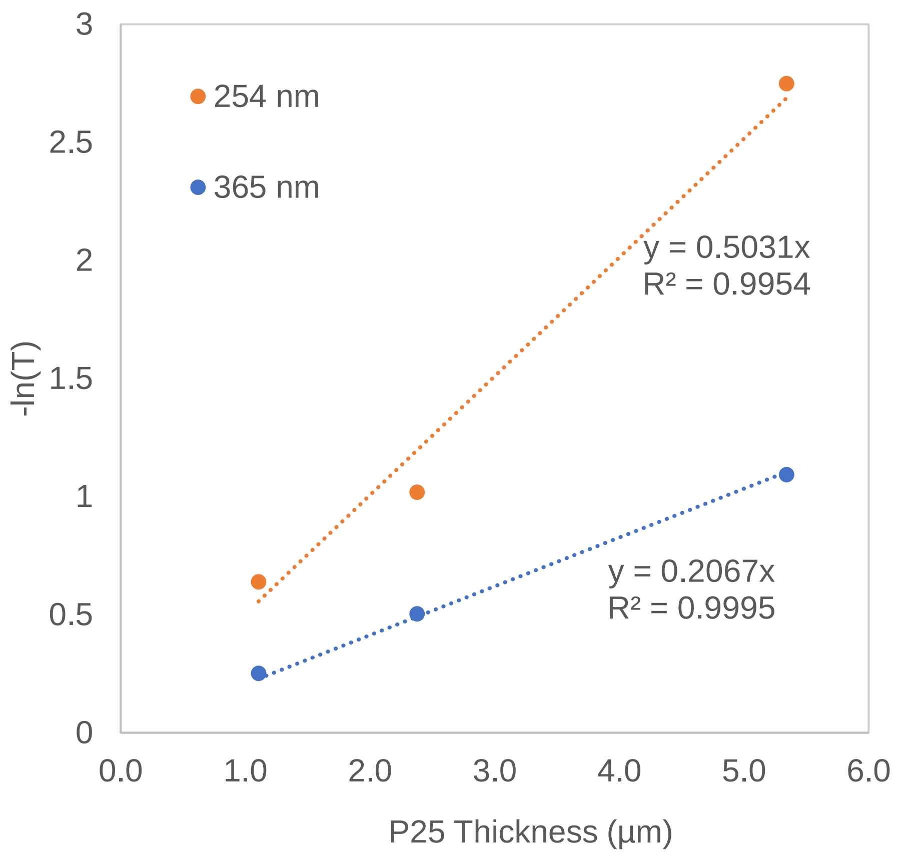

Figure S8. Plots of the porous P25 attenuation coefficient, α, at wavelengths of 254 nm and 365 nm, as derived from the slope of the relation between the negative natural logarithm of transmittance through the P25 films and the P25 film thickness across 3 different samples.

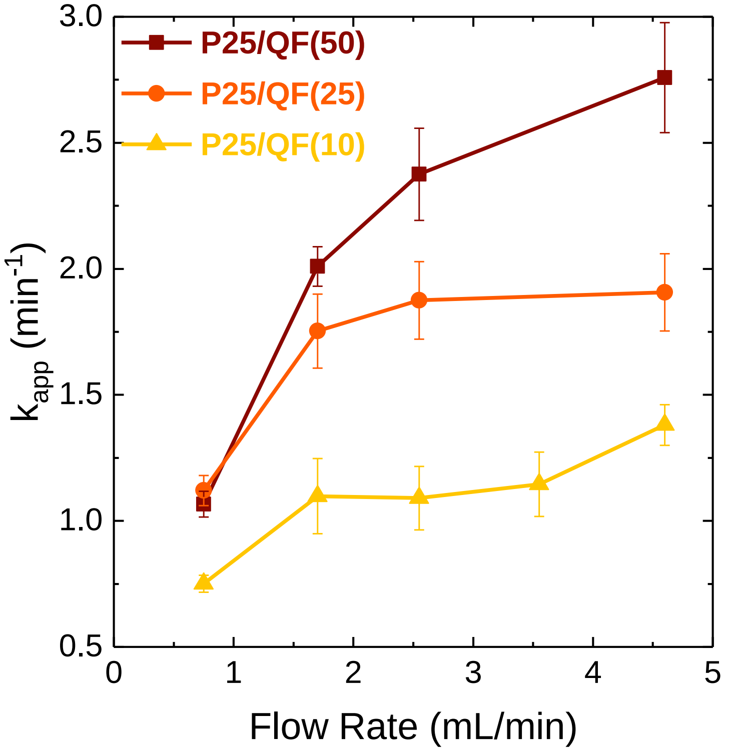

Figure S9. The apparent rate constant of RhB degradation for P25/QF(ρ) samples across multiple flow rates.

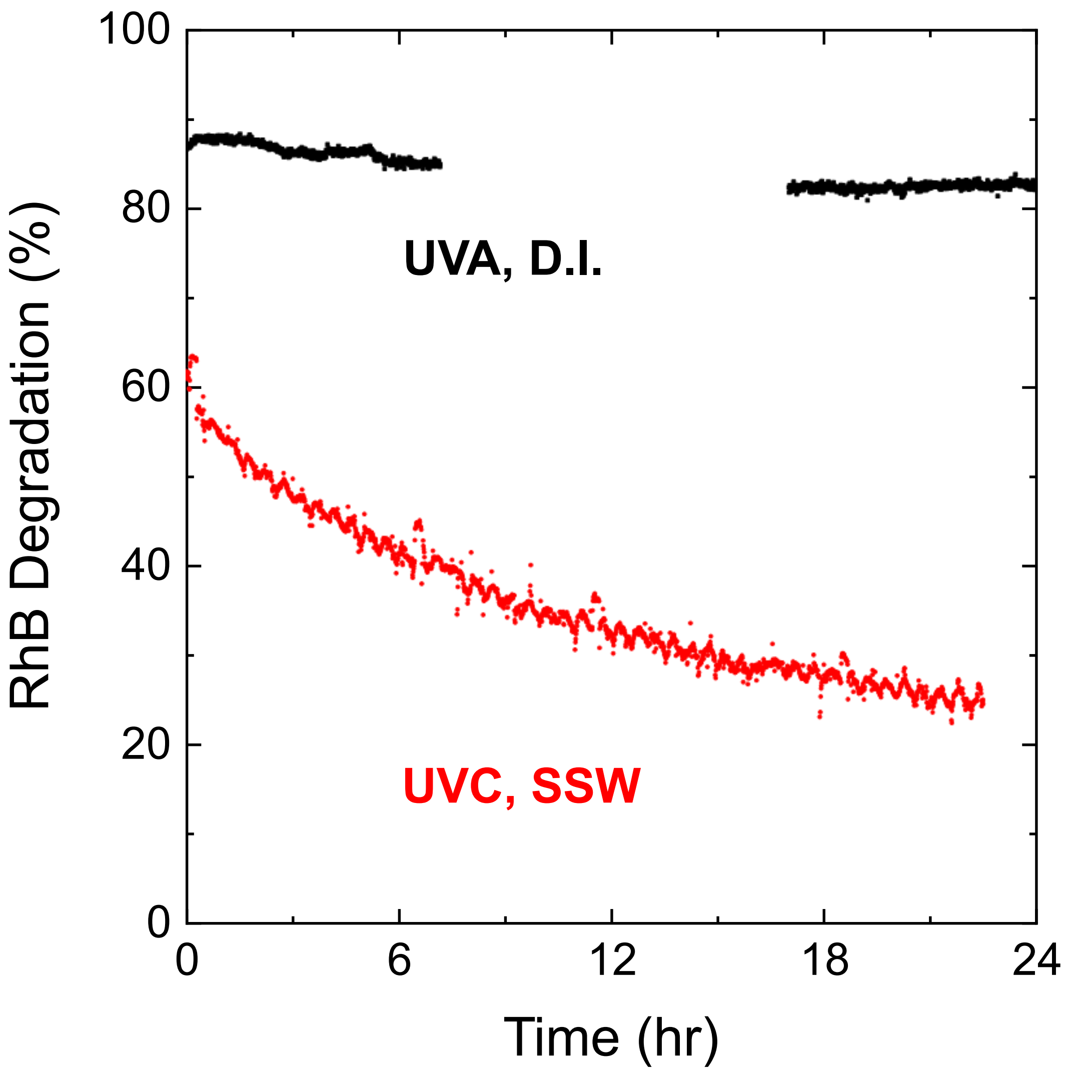

Figure S10. Long term degradation of RhB by P25/QF in DI water under UVA illumination at 0.75 mL/min flow and in SSW under UVC illumination at 4.6 mL/min. Note the discontinuity in the UVA, DI data is due to our fluorometer turning off during the overnight run. The run itself was continuous.

Additional Discussion:

Discussion S1. *Sample Properties & Model Calculations*

Table S1. Properties of P25/QF photocatalyst. P25/QF refers to the QF(50) density

Sample Properties	Value
QF Diameter (μm)	6.2 ± 1.2
QF Area Density (g/m^2)	10, 25, 50
P25/QF coating thickness (μm)	0.6
P25/QF photocatalyst loading (w/w %)	14
P25/QF mesoporosity (ε)	0.59
P25/QF Illuminated surface (m^2/m^3)	9.4 x 10^5
UVA Attenuation Coefficient, α_{365nm} (μm^{-1})	0.21
UVC Attenuation Coefficient, α_{254nm} (μm^{-1})	0.50

The QF Diameter was measured by analyzing optical microscopy images (5x to 50x magnification) of QF samples after removing the epoxy binder. The value provided is the average diameter from 7 images (± σ).

The QF Area Density is the mass of the QFs compared to the top-down, x-y surface area of the QF sample. The value is provided by the QF manufacturer (Saint-Gobain, Quartzveil™) and was verified empirically.

The P25 Thickness is the calculated mean thickness of porous P25 coating on the QF supports. To get to this value, we first calculated the overall surface area of quartz fibers in a given sample, then measured the mass of the P25 coating empirically, then calculated the thickness of a uniform shell coating on the cylindrical quartz fibers. This value was qualitatively validated through optical microscopy and SEM images.

The P25/QF Illuminated Surface is the ratio of P25 external surface area to the experimental reactor volume. The P25 wt (%) is the percentage of P25/QF sample mass

attributed to the P25 film alone. We measured the mass of P25/QF samples throughout the fabrication steps to arrive at this value.

The P25 mesoporosity (ε) is the calculated void fraction of porous P25 films. We used the ratio of the measured density of P25 films ($\rho_{film,}$ from side-view SEM) and the expected density of P25 powder ($\rho_{particle,}$ provided by Evonik) to calculate the mesoporosity via:

$$\varepsilon = 1 - \frac{\rho_{film}}{\rho_{particle}} \qquad (S1)$$

The attenuation coefficients at 254 nm illumination, α_{254nm} = 0.503 µm^{-1}, and at 365 nm, α_{365nm} = 0.207 µm^{-1}, were calculated from the Beer-Lambert law as applied to P25 films of thickness based on multiple coatings of P25:

$$\phi(x) = \phi_0 e^{-\alpha x} \qquad (S2)$$

$$\therefore \alpha_\lambda = -\frac{\ln(T_\lambda)}{x} \qquad (S3)$$

where T_λ is the transmittance of light with wavelength λ through a given thickness of P25/QF and x is the thickness of P25 film as calculated based on estimated and measured P25 film thickness distributed over a cross section of QF material (see Figure S8).

The mass of the QF after removal of the epoxy binder correlated well with the values provided by the manufacturer (Saint Gobain). Using this data and the density of quartz (2.67 g/cm^3), we calculated the QF density and microporous void fraction. Having measured a QF fiber diameter of 6.2 (± 1.2) µm through optical microscopy (and supported by x-ray tomography), we calculated the number of QF layers vs. QF density, QF surface area, and QF volume.

A variety of properties of the P25 catalyst were measured empirically. First, we measured the mass of samples at all steps of the fabrication process to calculate the P25 film mass. This was compared to the density of P25 TiO_2 (Evonik) with equation (1) to calculate the mesoporous

void fraction. The P25 catalyst loading was calculated based on the mass % of P25 film to QF fiber.

Discussion S2*. Mass Transport Modeling*

From Section 3.3:

We formed a basic theoretical framework to understand the comparative performance of the photocatalyst and to build upon for scaling-up to larger water treatment systems. We proposed several parameters which influence the system: 1) fluid flow profile, 2) catalyst loading and surface area, 3) internal mass transport, 4) external mass transport, 5) wavelength of UV excitation, and 6) UV intensity.

We established a residence time of bulk fluid through our reactor through a series of pulsed dye tests (Figure S5). The results showed near plug flow profiles for systems with and without QF, though the presence of QF(50) increased the axial dispersion of our system from 0.028 cm^2/s to 0.036 cm^2/s which we account for through the change in Bodenstein number from a value of 30.3 without QF to 23.7 with QF. The decrease in Bodenstein number corresponds to an increase in back mixing in our system. We further described the flow in our system through Reynold's number, which was calculated to be 3.07 for the non-QF system at 4.6 mL/min flow and 0.49 for the QF system at same flow using a modified Reynold's number for flow through porous media. The transition to turbulent flow happens at different values for traditional and modified Reynold's numbers ($Re > 2000$, and $Re > 10$, respectively), so the relative turbulence of the QF and non-QF systems is difficult to assess but the flow in both systems is clearly laminar. Using this Reynold's number, and the Schmidt number (ν/D_{ab}), we also approximate the external mass transfer coefficients through a set of mass transfer correlations which quantifiably demonstrates one area in which the QF system yields performance benefits over a flat catalyst substrate (Table S3).

Both mass and optical transport in our photocatalytic system are impacted by the total loading of P25 within the reaction chamber. For P25/QF(50), the catalyst loading is ~18.6 g/L, much higher than many other ratios reported in literature.[7] Previous studies have suggested that suspended TiO_2 catalyst loads of <2 g/L are ideal from an efficiency standpoint, wherein higher suspended catalyst loads prevent optical transport through the medium.[12, 27, 34] This light scavenging effect may help explain the higher quantum yield of P25/QF(10), where the TiO_2 loading is ~3.7 g/L, vs. P25/QF(50).

As seen in the prior section, P25/QF systems having a lower catalytic loading with respect to the MFR volume achieved higher photonic efficiencies. One way to assess this information is to consider the optical path length through each P25/QF loading.[35] Here, the optical path length is defined as the depth through which UV illumination can penetrate before being attenuated e-fold by the P25/QF at a given density. This calculation is based on the Beer-Lambert law, provided in equation (5). We normalized the absorption coefficients of P25/QF(ρ) samples to the fixed depth of the MFR (500 µm) and back-calculated the optical path lengths, provided in Table S3. Note that the P25/glass sample was evaluated at the fixed P25 film thickness of 5 µm. As the P25/QF becomes less dense with respect to the reactor volume, the penetration depth of UV light increases. Therefore, when attempting to optimize and scale up this P25/QF system, engineers could target a higher optical path length, or lower catalytic loading density, to improve the overall energy efficiency of the system and lower operation costs.

Table S3. Characteristics and Performance Metrics for Photocatalysts Under UVA.

Sample	Axial Dispersion (cm^2/s)	Specific Surface Area (m^2/g)	Catalyst Loading (mg)	Est. Mass Transfer Coefficient (cm/s, x 10^{-4})	UVA Optical Path Length (μm)	Quantum Efficiency (%)	RhB Deg UVA E_{EO} (kWh/m^3)
**P25/glass**	0.028	-	4.68	6.35	4	0.46	72.6
**P25/QF(10)**	-	-	4.7	12.6	2012	2.47	1.94
**P25/QF(25)**	-	-	10.1	12.6	979	1.88	1.41
**P25/QF(50)**	0.036	8.22	22.7	12.6	382	1.39	0.90

As mentioned in Section 3.1, we established residence time of bulk fluid through our reactor through a series of pulsed dye tests. The results (Figure S5) showed near plug flow profiles for systems with and without QF, though the presence of QF(50) increased the axial dispersion of our system. That is, some minor back-mixing is present in QF systems and can be accounted for through the Bodenstein number (or Peclet number) in future models:

$$Bo = \frac{uL}{D_{ax}} \tag{S4}$$

To further describe the flow in our system, we calculate the Reynold's number (i.e., the dimensionless ratio of the inertial to viscous forces of a fluid):

$$Re = \frac{uL}{\nu} \tag{S5}$$

Where u is the velocity of the fluid, ν is the fluid kinematic viscosity (dynamic viscosity divided by density) and L is the characteristic length scale of the system. Determination of the L term

depends primarily on the system geometry. For rectangular channels (such as in our MFR), L can be described by the hydraulic diameter:

$$L = D_{H,channel} = 4R_H = \frac{4A}{P} = \frac{4WH}{2(W+H)}$$
$$For\ W \gg H \rightarrow\ D_{H,channel} \approx 2H \qquad (S6)$$

The height of the MFR channel is 0.05 cm; thus for a fluid flow of 4.6 mL/min (= 0.307 cm/s) and a kinematic viscosity of water at room temperature of ~0.01 cm^2/s, the Reynold's number through our open channel is equal to 3.07. This is squarely within the regime of laminar fluid flow.

For flow through a porous system (as in our QF), we use a modified Reynold's number which accounts for porosity of the medium:

$$Re_i = \frac{u_p D_F \phi}{\nu(1-\phi)} = \frac{u_s D_F}{\nu(1-\phi)} \qquad (S7)$$

Where $u_p$is the flow velocity within the porous structure and u_s is the superficial velocity that would be experienced if there were no porous medium. For our system, where the diameter of our fibers (D_F) is in the range of 6.2x10^{-4} cm and the porosity is ~0.95, the modified Reynold's number is ~0.49. While the modified Reynold's number is lower in porous media, the regimes of laminar vs turbulent flow changes in relation to Re and Re_i. For Re, laminar flow can usually be approximated by Re < 2000 whereas with Re_i, laminar flow is typically distinguished by < 10. There is no hard rule for describing the laminar regime since the makeup of porous structures can change significantly; nevertheless, the Re and Re_i for our systems indicate laminar flow.[1]

One useful aspect of the Reynold's number is that it can be used in combination with the Schmidt number (ν/D_{ab}) to approximate the mass transfer coefficient of a fluid system. That is, $Sh = \oint(Re, Sc) = k_m\, L/D_{ab}$. The functional relation between Sh and Re & Sc, however, is strongly dependent on specific system properties. This so-called mass transfer correlation has been studied for a variety of systems. For our open MFR channel, a simple relation can be taken as

$$Sh = \frac{k_m L}{D_{ab}} = 0.646\,(Re)^{\frac{1}{2}}\,(Sc)^{\frac{1}{3}} \tag{S8}$$

This is a commonly used mass transport correlation for pipes and simple open systems. For porous flow, this relation can vary dramatically. A few potentially suitable relations are provided below. In all cases, the mass transport coefficient, k_m, is itself a function of the fluid velocity, u; thus, unsurprisingly, the faster the fluid flows the greater the mass transport in a system. Note too that the functional relation may not always be intimately tied to the Re or Sc, but rather to a dimensionless makeup of related variables. In this case too, the modified Reynold's number may have a different impact on the mass transport given the porous system of choice, much like in the case of determining laminar vs turbulence of flow.

For packed-bed systems, the mass transport is often correlated to the flow through the Chilton-Colburn J-factor:[2]

$$J_D = \frac{k_m Sc^{\frac{2}{3}}}{u} = \frac{1.09}{\varepsilon Re^{0.67}} \tag{S9}$$

Which yields similar results to another mass transfer correlation:[3]

$$\frac{k_m}{u} = 1.17(Re)^{-0.42}\left(\frac{1}{Sc}\right)^{\frac{2}{3}} \tag{S10}$$

Within porous catalysts, the internal kinetic considerations are diffusion and reaction [4-5] Given a 2D slab of porous catalyst, the general equation reduces to:

$$D_{eff}\frac{\partial^2 c}{\partial y^2} - r = 0 \tag{S11}$$

Here, the diffusion within the catalyst is described as an effective diffusion and is a function of the catalyst porosity (ε) and tortuosity (τ) in addition to the standard diffusion coefficient for a solute (D_{ab}) as

$$D_{eff} = D_{ab}\frac{\epsilon}{\tau} \qquad (S12)$$

For many soluble species in water at 25 °C, the diffusion coefficients are on the order of 10^{-5} or 10^{-6} cm^2/s.[3] For Rhodamine B, Gendron et. al. measured the diffusion coefficient in water to be 4.2 $x10^{-10}$ m^2/s, which is the value we use throughout this study.[6] For the tortuosity, we assume isotropic arrangement of the P25 particles and apply the Bruggman tortuosity approximation of $\tau = \epsilon^{-\frac{1}{2}}$ yielding a tortuosity of ~ 1.30.[7] With this value, we estimated the effective internal diffusion of aqueous RhB within our porous P25 catalyst as D_{eff} = ~ 1.85 x 10^{-6} cm^2/s.

Using our calculated axial diffusion, mass transport, and effective diffusion as well as our measured catalyst loading and apparent reaction rates, we can implement these into a constitutive equation for one-dimensional steady-state flow:

$$D_{ab}\frac{d^2C_A}{dz^2} - U_z\frac{dC_A}{dz} + r_A = 0 \qquad (S13)$$

Which describes both diffusion (in the D_{ab} term) and convection (driven by U_z) along with the effective reaction given through r. This sort of 1D relation is most valid for plug flow reactors, where the concentration of species in the bulk does not change considerably in the x or y directions. Equation (S13) is also applicable to reactors in which there is dispersion, where D_{ax} takes the place of the diffusion term, D_{ab}.

The reaction term in the governing equation (S11) must be considered now for the degradation of RhB within our system. We first assume that the breakdown of RhB is primarily driven by the oxidation by •OH in solution. This assumption is based on 1) the high oxidation potential of •OH, 2) the high concentration of •OH in the MFR, i.e. 72 μM, and 3) we are operating at or near the point of zero charge for P25 resulting in minimal electrostatic adsorption. Given a steady-state generation of •OH by the UV-P25 system, we can approximate the degradation of

RhB to be first-order with respect to RhB concentration. Thus, $r = k' C_{RhB}$ where k' is the apparent rate constant (itself dependent on numerous factors including flow rate & illumination intensity).

Discussion S3. *Defense of Using a Terephthalic Acid Probe under UVC*

Excitation of TPA/hTPA systems with UVC requires care because both TPA and hTPA are photosensitive. Previous studies have implemented TPA under UVC illumination for •OH quantification.[8]

For our work, we conducted batch tests of UVC interactions with TPA/hTPA systems to examine and isolate potential impacts. First, we illuminated catalysts immersed in TPA solutions under UVC and collected samples every 5 min to examine the hTPA resulting fluorescence (excitation = 350 nm, emission = 425 nm). Our initial batch test of P25/QF catalyst, conducted in a 25 μM solution of TPA, showed an initial spike in hTPA emission, followed by a steady decrease (Figure S11). Hypothesizing this to be from full consumption of TPA reactant, we re-ran the test of P25/QF in a 100 μM TPA solution and observed a clearer trend. Herein, we observed an initial linear rise in hTPA and followed by a leveling and decline in fluorescence (Figure S12) attributed to the degradation of hTPA through photolytic and •OH-mediated mechanisms.

`

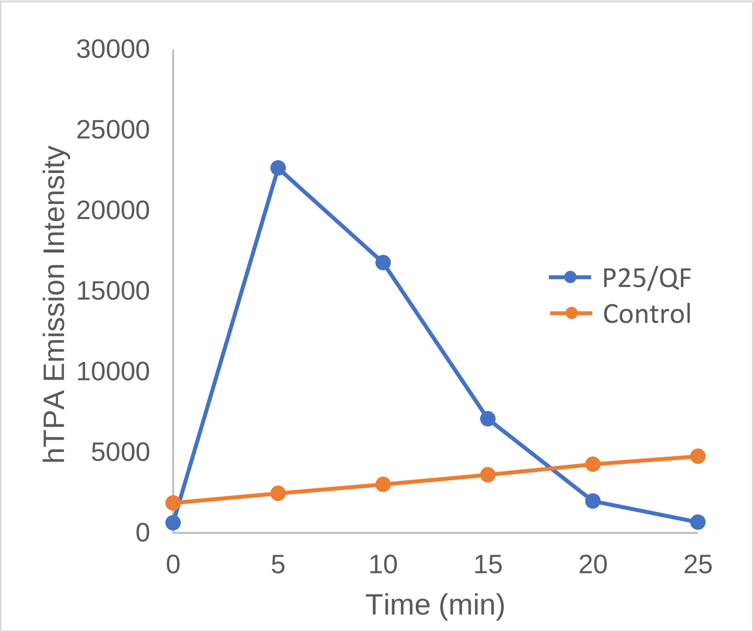

Figure S11. Accumulation of hTPA over time for P25/QF catalyst. The control is in the absence of catalyst.

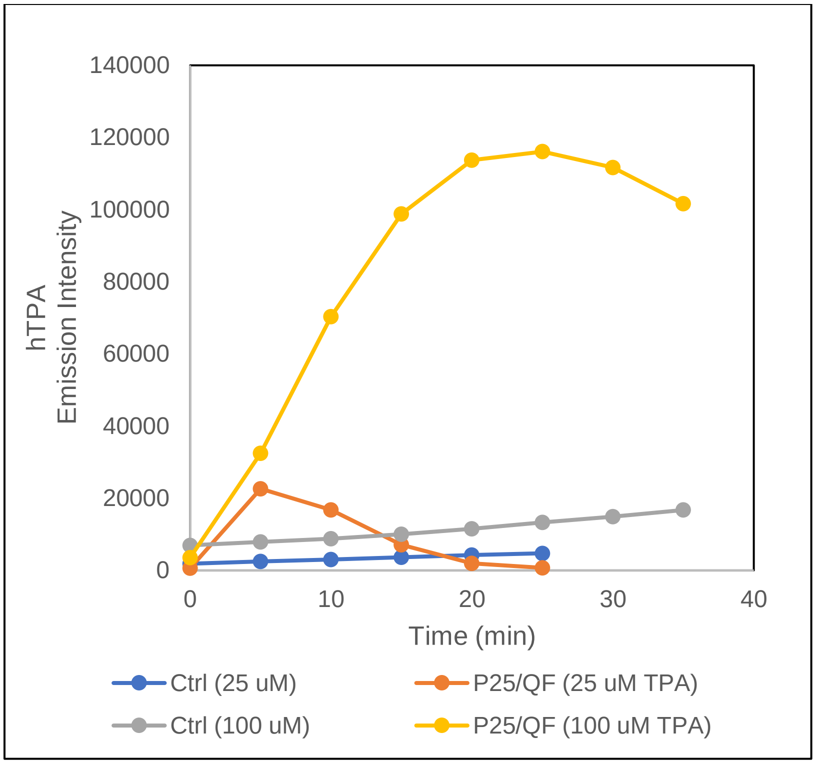

Figure S12. Accumulation of hTPA over time for P25/QF catalysts in 25 μM and 100 μM solutions of TPA, along with their no-catalyst controls.

We can explain the potential photonic effects on hTPA by its generation and degradation as follows:

$$\frac{d[hTPA]}{dt} = r_1 + r_2 - r_3 - r_4 \qquad (A1)$$

Where:

- r_1 is the generation of hTPA from direct photonic interactions with TPA
- r_2 is the generation of hTPA from reaction between TPA and •OH
- r_3 is the destruction of hTPA from direct photonic interaction
- r_4 is the destruction of hTPA from the reaction between hTPA and •OH

We conducted batch tests to examine r_1 and r_3 directly, with results shown in Figure S13a and S13b, respectively.

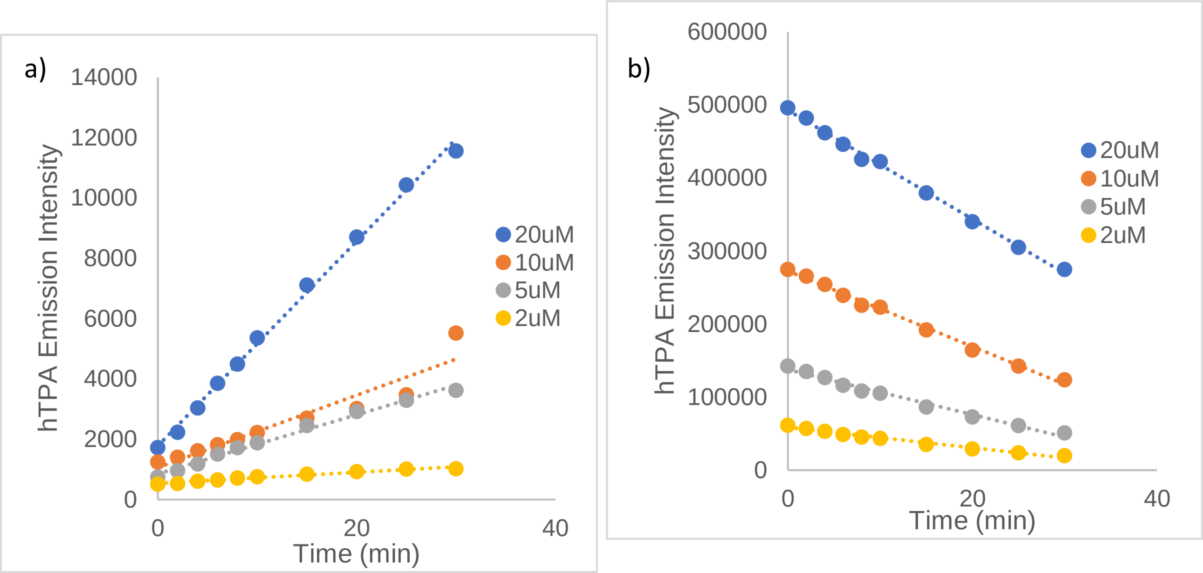

Figure S13. a) The generation of hTPA from UVC/TPA interactions at different initial [TPA]. b) The UVC-driven degradation of hTPA at various initial concentrations of [hTPA].

While $r_3 > r_1$ at equivalent concentrations of the reactant (TPA and hTPA, respectively), the rate of hTPA generation from low initial levels of TPA always increased in no-catalyst control tests (see control data in Figures S11 & S12). The rates r_1 and r_3 appear linear and dependent upon the initial reactant concentration. However, the impacts of r_1 and r_3 on the overall change in [hTPA] are largely outweighed by hTPA generation through rate r_2 in catalyzed systems. This hydroxyl-mediated generation of hTPA can be described by the following pseudo-first-order reaction:[8]

$$r_2 = k_{TPA}[TPA][\cdot OH]_{SS}Y_{hTPA} \tag{A2}$$

Where k_{TPA} is the second-order reaction rate constant of TPA and •OH (reported as $4.4x10^9$ M^{-1} s^{-1} in Page *et al.*[9]), and $[•OH]_{SS}$ is the steady-state concentration of hydroxyl radical, and Y_{hTPA} is the molar yield of hTPA from the reaction of TPA and •OH (reported as 0.35 by Page *et al.*[9]).

In tests reported in the main body of our work, we further isolated the •OH generation through r_2 by

1) reacting TPA in excess (i.e., TPA >> hTPA, generally a $[TPA]_0$ = 500 μM) so that the formation of hTPA largely outweighed its degradation

2) implementing flow-through systems, which limited the relative consumption of TPA and impact of increasing levels of hTPA

Through these exploratory tests and careful experimentation, we could minimize the impact of r_1, r_3, and r_4 to better approximate the generation of •OH through hTPA generation attributed to the rate r_2. To the extent that our findings may deviate from actual •OH generation, we expect our results to underestimate •OH generation owing to the fact that increasing $[•OH]_{SS}$ also increases the degradation rate r_4, which is not controlled for within this study.

The data for •OH formation as measured through the hTPA probe is also supported by the applied data for RhB and micropollutant degradation in the main body of work.

References

1). Dwivedi, P. N.; Upadhyay, S. N., Particle-Fluid Mass Transfer in Fixed and Fluidized Beds. *Industrial & Engineering Chemistry Process Design and Development* **1977,** *16* (2), 157-165.
2). Wilson, E. J.; Geankoplis, C. J., Liquid Mass Transfer at Very Low Reynolds Numbers in Packed Beds. *Industrial & Engineering Chemistry Fundamentals* **1966,** *5* (1), 9-14.
3). Cussler, E. L. In *Diffusion: Fundamentals of Mass Transfer*, 2009.
4). Fogler, H. S., *Elements of chemical reaction engineering*. Third edition. Upper Saddle River, N.J. : Prentice Hall PTR, [1999] ©1999: 1999.
5). Visan, A.; van Ommen, J. R.; Kreutzer, M. T.; Lammertink, R. G. H., Photocatalytic Reactor Design: Guidelines for Kinetic Investigation. *Industrial & Engineering Chemistry Research* **2019,** *58* (14), 5349-5357.

6). Gendron, P. O.; Avaltroni, F.; Wilkinson, K. J., Diffusion Coefficients of Several Rhodamine Derivatives as Determined by Pulsed Field Gradient–Nuclear Magnetic Resonance and Fluorescence Correlation Spectroscopy. *Journal of Fluorescence* **2008,** *18* (6), 1093-1101.
7). Tjaden, B.; Cooper, S. J.; Brett, D. J. L.; Kramer, D.; Shearing, P. R., On the origin and application of the Bruggeman correlation for analysing transport phenomena in electrochemical systems. *Current Opinion in Chemical Engineering* **2016,** *12*, 44-51.
8). Zhang, G.; Wei, S.; Wu, B.; Chen, Z.; Zhang, S., Nonnegligible Generation of Hydroxyl Radicals from UVC Photolysis of Aqueous Nitrous Oxide. *Environmental Science & Technology* **2018,** *52* (17), 9785-9792.
9). Page, S. E.; Arnold, W. A.; McNeill, K., Terephthalate as a probe for photochemically generated hydroxyl radical. *Journal of Environmental Monitoring* **2010,** *12* (9), 1658-1665.
